# Cystatin F is a biomarker of prion pathogenesis in mice

**DOI:** 10.1371/journal.pone.0171923

**Published:** 2017-02-08

**Authors:** Mario Nuvolone, Nicolas Schmid, Gino Miele, Silvia Sorce, Rita Moos, Christian Schori, Roger R. Beerli, Monika Bauer, Philippe Saudan, Klaus Dietmeier, Ingolf Lachmann, Michael Linnebank, Roland Martin, Ulf Kallweit, Veronika Kana, Elisabeth J. Rushing, Herbert Budka, Adriano Aguzzi

**Affiliations:** 1 Institute of Neuropathology, University Hospital of Zurich, Zurich, Switzerland; 2 Cytos Biotechnology AG, Zurich-Schlieren, Switzerland; 3 AJ Roboscreen GmbH, Leipzig, Germany; 4 Department of Neurology, University Hospital Zurich, Zurich, Switzerland; 5 Department of Neurology; Bern University Hospital and University of Bern, Bern, Switzerland; Nagasaki University Graduate School of Biomedical Sciences, JAPAN

## Abstract

Misfolding of the cellular prion protein (PrP^C^) into the scrapie prion protein (PrP^Sc^) results in progressive, fatal, transmissible neurodegenerative conditions termed prion diseases. Experimental and epidemiological evidence point toward a protracted, clinically silent phase in prion diseases, yet there is no diagnostic test capable of identifying asymptomatic individuals incubating prions. In an effort to identify early biomarkers of prion diseases, we have compared global transcriptional profiles in brains from pre-symptomatic prion-infected mice and controls. We identified *Cst7*, which encodes cystatin F, as the most strongly upregulated transcript in this model. Early and robust upregulation of *Cst7* mRNA levels and of its cognate protein was validated in additional mouse models of prion disease. Surprisingly, we found no significant increase in cystatin F levels in both cerebrospinal fluid or brain parenchyma of patients with Creutzfeldt-Jakob disease compared to Alzheimer’s disease or non-demented controls. Our results validate cystatin F as a useful biomarker of early pathogenesis in experimental models of prion disease, and point to unexpected species-specific differences in the transcriptional responses to prion infections.

## Introduction

Prion diseases or transmissible spongiform encephalopathies are rare, late-onset, rapidly progressing and fatal infectious neurodegenerative disorders [1]. They include sporadic, genetic, variant or iatrogenic Creutzfeldt-Jakob disease (CJD) in humans, ovine scrapie, bovine spongiform encephalopathy, and chronic wasting disease of deer and elk [1]. The etiologic agent is termed prion and consists of the scrapie prion protein (PrP^Sc^), a misfolded, aggregated, often partially protease-resistant conformer of the cellular prion protein (PrP^C^) [2]. PrP^Sc^ is postulated to exist in different conformations, corresponding to different prion strains, which differ in their biochemical properties, and cause different forms of the disease in susceptible host species [3]. Misfolding of PrP^C^ into PrP^Sc^ and accumulation of PrP^Sc^ aggregates within the central nervous system (CNS) result in progressive neuronal loss and pathognomonic spongiform vacuolation, accompanied by intense astro- and microgliosis [4].

Neuroimaging and neuro-electrophysiological tests, analysis of 14-3-3 protein and other surrogate biomarkers of CNS damage in the cerebrospinal fluid (CSF) and, more recently, the demonstration of PrP^Sc^ seeding activity in CSF or nasal brushings, can support the diagnosis of prion disease in the presence of a consistent clinical picture[5]. However, a definitive diagnosis requires the demonstration of PrP^Sc^ in brain tissue, most often performed *post-mortem* with immunohistochemical-based techniques [5].

Experimental transmission, as well as acquired and iatrogenic cases of prion disease after known exposure to prions, indicate that prion infection undergoes a long incubation before its clinical onset [1]. Identification of patients with prion disease during the pre-clinical stage could be instrumental in minimizing the risk of iatrogenic diseases (for example by deferral of blood donors), and is a precondition to early intervention before the establishment of more advanced neurodegeneration once effective therapies will become available. Disappointingly, the development of validated diagnostic tests to identify pre-symptomatic patients with prion disease still remains an unmet medical need.

In an effort to identify novel biomarkers of prion diseases, we performed a global, microarray-based transcriptomic analysis in brains of pre-symptomatic, prion-infected mice [6]. This led to the identification of the serine/cysteine protease inhibitor *Serpina3n* as an early upregulated transcript during prion disease, and of urinary levels of its human homologue, α1-antichimotrypsin as a surrogate biomarker of prion infection [6]. In the present study, we have re-analyzed this microarray dataset using alternative computational methods to uncover additional candidate biomarkers.

## Materials and methods

### Ethical statement

Animal care and experimental protocols were in accordance with the “Swiss Ethical Principles and Guidelines for Experiments on Animals” and with the Swiss Animal Protection Law, and approved by the Veterinary office of the Canton Zurich (permits 85/2003, 171/2004, 30/2005, 41/2012 and 90/2013). All efforts were made to minimise animal discomfort and suffering.

All human tissue samples used in this project were irreversibly anonymized and collected before the year 2005. Approval by an Institutional Review Board is not required for the use of irreversibly anonymized samples collected before the approval of the Swiss Medical-ethical guidelines and recommendations (Senate of the Swiss Academy of Medical Sciences, Basel, Switzerland, May 23^rd^ 2006). The study using human CSF was approved by the Swiss Ethics Committees of Canton Zurich (KEK-ZH-Nr. 2012–0376, Amendment dt. 05.07.2014).

### Patient populations

#### Cohort 1

This cohort comprised CSF samples from cases with different neuroinfectious, neuroinflammatory and neurodegenerative conditions. Leftovers from diagnostic CSF samples were obtained from the biobank of the Department of Neurology, University Hospital Zurich. Clinical data concerning diagnosis, gender, date of birth/death and lumbar puncture (LP) were collected from medical charts and autopsy reports obtained from the hospital’s databases PathoPRO and KISIM.

After the performance of desired diagnostic tests, CSF samples were spun down at 3000 rpm for 10 min, aliquoted and since then stored at -80° C.

#### Cohort 2

This cohort comprised CSF samples from cases with autopsy-proven, definitive Creutzfeldt-Jakob disease or Alzheimer’s disease, as well as cases with clinically diagnosed Alzheimer’s disease and from non-demented, control subjects.

Leftovers from diagnostic CSF samples from subjects with autopsy-proven, definitive Creutzfeldt-Jakob disease (Def CJD, n = 39) or Alzheimer’s disease (Def AD, n = 11) were obtained from the CSF biobank of the Swiss Referral Center for Prion Diseases (Nationales Referenzzentrum für Prionenerkrankungen, NRPE, located at the Institute of Neuropathology, University Hospital Zurich). The NRPE is in charge of the registration and surveillance of human transmissible spongiform encephalopathies in Switzerland. It provides, *inter alia*, p14-3-3 detection in CSF and autopsies of possible or probable CJD patients from all over the country. CSF samples from the NRPE-CSF biobank were sent ideally on dry ice from corresponding hospitals for p14-3-3 detection, temporarily kept at -20°C awaiting Western blot analysis and were finally stored at -80° C.

Definitive diagnosis of Creutzfeldt-Jakob disease was made by a board certified neuropathologist, according to current guidelines after histopathological examination and the detection of proteinase K resistant PrP^sc^ in brain homogenates by Western blot analysis. Neuropathologic assessment of Alzheimer type brain pathology was made according to the NIA/AA criteria [7]. For all NRPE cases, date of CSF draw, date of death and autopsy, histopathological report and p14-3-3 results were available. Samples obtained from consecutive cases evaluated between 2004 and 2014 were included in this cohort. Cases with insufficient CSF leftover or with macroscopically haemorrhagic CSF (14 DEF CJD and 2 Def AD) were excluded from the study.

Leftovers from diagnostic CSF samples from subjects with clinically diagnosed Alzheimer’s disease (Clin AD, n = 26) and from non-demented, control subjects (Controls, n = 24) were obtained from the biobank of the Department of Neurology, University Hospital Zurich.

The clinical history of Alzheimer’s disease cases was extensively assessed and diagnosed according to strictly applied diagnostic criteria. Patients underwent a cranial MRI scan (all), neuropsychological evaluation (all), EEG (n = 22), Mini Mental Status (n = 24), CSF testing (all, n = 11 with neurodegenerative markers such as p-tau and amyloid β). Twenty-five patients had at least one additional follow up evaluation at the Clinic of Neurology after CSF withdrawal, with the clinical picture being still compatible with a diagnosis of AD. In three cases the clinical history was compatible with a diagnosis of mixed dementia caused by the coexistence of neurodegenerative and vascular changes. The group of clin AD was matched to the def AD and def CJD groups in terms of patients’ age and gender and duration of sample storage.

The non-demented, control group (controls, n = 24) was composed of patients who underwent clinical examination including lumbar puncture during the work-up of one of the following symptoms: headache, vertigo, sleep disorder. Fourteen patients underwent a cranial MRI showing no pathological changes. All patients received at least two clinical evaluations at different time points and no other neurological complaints including signs of cognitive impairment or neurodegeneration were reported. The control group was matched to the others based on the gender distribution and on the duration of sample storage.

#### Cohort 3

Archival brain tissue from patients with autopsy-proven, definitive Creutzfeldt-Jakob disease (n = 11) or Alzheimer’s disease (n = 8) were obtained from the autopsy biobank of the NRPE at the Institute of Neuropathology, University Hospital Zurich.

### Mice

C57BL/6J male mice were purchased from Charles River. Mice were kept in a conventional hygienic-grade facility, housed in groups of 3–5 in type IIL cages under a 12 h light/dark cycle (light from 7 am to 7 pm) at 22±1°C, with unrestricted access to sterilized food (Kliba No. 3340, Provimi Kliba, Kaiseraugst, Switzerland) and water.

#### Prion inoculation

Mice were anesthetized with isofluorane and injected in the right hemisphere with 30 μl of 0.1% of RML6 (passage 6 of Rocky Mountain Laboratory strain mouse-adapted scrapie prions) or of 0.1% of non-infectious brain homogenate (NBH) from CD-1 mice as control [8]. Mice were monitored three times per week in the absence of any clinical sign of disease and every day after clinical onset. Clinical assessment and scoring were performed as previously described [9] with minor modifications (S1 Table). Actions were taken to minimize animal distress and suffering (S1 Table). Prion-infected mice were sacrificed at 27, 56, 82, 110, 123 and 137 days post-inoculation (dpi) or when they reached the terminal stage of prion disease at ca. 176 dpi. One additional group of control mice injected with NBH was sacrificed one week after the last prion-injected mouse reached the terminal stage, at 192 dpi. Euthanasia was performed through transcardial perfusion with PBS after deep anesthesia with ketamine and xylazine (*N*-(2,6-Dimethylphenyl)-5,6-dihydro-4*H*-1,3-thiazin-2-amine). Brain areas were dissected, snap frozen and kept at -80°C until further processing. Archival murine CNS samples from mice inoculated with RML and 22L rodent-adapted scrapie prions and control NBH-injected mice from previously published studies [6,8,10] were analyzed.

#### Experimental autoimmune encephalitis

Experimental autoimmune encephalitis was induced in 13–16 week old C57BL/6 mice through subcutaneous administration of 200 μg of MOG_35-55_ peptide (MEVGWYRSPFSRVVHLYRNGK; Neosystem, Strasbourg, France) emulsified in complete Freund’s adjuvant (CFA) supplemented with 4 mg/ml Mycobacterium tuberculosis (DIFCO, Detroit, MI, USA), as previously described [11]. Mice received intraperitoneal injections with 200 ng pertussis toxin (Sigma, Switzerland) at the time of immunization and 48 hours later. Control mice received an identical regimen with the exception of MOG_35-55_ peptide. Clinical assessment and scoring were performed as previously described [11] and actions were taken to minimize animal distress and suffering (S2 Table). Mice with experimental autoimmune encephalitis and control mice were sacrificed in parallel.

Genetically modified mice used in this study are listed in Table 1.

**Table 1:** Genetically modified mice used in this study.

Line (abbreviation)	Genetic modification	Description of mice used	Ref.
***Prnp*^ΔF^ (ΔF)**	Transgenic mice expressing a truncated PrP^C^ (32–134) on a B6129-*Prnp*^ZrchI/ZrchI^ background	Adult mice with signs of neurodegeneration	[31]
**B6-Tg/Thy1APP23Sdz (APP23)**	Transgenic mice overexpressing the human APP with the Swedish double mutation at 670/671 (KM→NL)	1.5 year-old mice with histologic evidence of astrogliosis, microgliosis and amyloid β plaque	[32]
**C57BL/6J-*Prnp*^ZH3/ZH3^ (ZH3)**	Co-isogenic C57BL/6J mice lacking the cellular prion protein.	Adult mice	[18]

### Reverse transcription and polymerase chain reaction

Total RNA was isolated using Trizol (Invitrogen AG, Switzerland) or the RNeasy Universal Plus Mini kit (Qiagen). Prior to cDNA synthesis, residual genomic DNA was removed by the DNA free-kit (Ambion, USA) or using the gDNA Wipeout buffer (Qiagen). cDNA was synthesized using the bulk first strand cDNA synthesis kit and *Not* I-(dT)_18_ as primer (Amersham Biosciences Europe GmbH, Germany) or with Quantiscript Reverse Transcriptase (Qiagen). Control reactions omitting the reverse transcriptase enzyme were performed to verify successful removal of genomic DNA. All these procedures were performed according to the manufacturers’ protocols. Extracted RNA was analysed using a ND-1000 Spectrophotometer (NanoDrop).

Primer pairs were designed using Primer3 software (http://frodo.wi.mit.edu/cgi-bin/primer3/primer3_www.cgi) or using NCBI primer blast (http://www.ncbi.nlm.nih.gov/tools/primer-blast/) or derived from [12].

For amplification we used the SYBR Green PCR Master Mix (Qiagen) or the Fast Start SYBR Green Master Mix (Roche) with 0.3–1 uM of each forward and reverse primers for the target of interest and cDNA (or no reverse transcriptase sample or water, as controls) as template.

The reaction was run on an ABI PRISM 7700 Sequence Detection System (Applied Biosystems) or on a ViiA 7 Real-Time PCR System (Life Technology) using default cycle conditions followed by a melting curve analysis. Samples were tested in technical triplicates. Expression of a subset of transcripts identified by microarray as upregulated during prion disease (which also included *Cst7*) was measured in mouse brain and normalized to *Actb* levels. Expression of *Cst7* in different mouse organs was normalized by geNorm [13] to the levels of the following normalization genes: *Gapdh*, *Eif2a*, *Utp6c*, *Hprt1*.

### Northern blotting

Total RNA was isolated using Trizol (Invitrogen AG, Switzerland) according to standard procedures. Northern blotting was performed exactly as previously described[6]. Northern blotting for *Cst7* was performed in parallel to Northern blotting for *Serpina3n*, *Gfap* and *Rn18s* as reported in [6] and hence blots for *Serpina3n*, *Gfap* and *Rn18s* are reproduced from [6].

Murine cDNAs for derivation of *Cst7* probe was obtained from Open Biosystems (www.openbiosystems.com), accession number AA089317.

Uniform adjustment of contrast and levels on the acquired images and subsequent cropping were performed using Adobe Photoshop. Unprocessed Northern and Western blot images are provided in S1 File.

### RNA sequencing

Data on *Cst7* mRNA expression levels in different cell types of the mouse brain were retrieved from the RNA-sequencing-based transcriptome database for mouse brain cortex cells [14]. Data on *CST7* mRNA expression levels in different cell types of the human brain were retrieved from the RNA-sequencing-based transcriptome database for human brain cortex cells [15]. Data on *Cst7* mRNA expression levels during microglia development and upon LPS stimulation were retrieved from an RNA-sequencing-based transcriptome database for mouse microglia cells [16]. RNA sequencing on hippocampi and cerebella of prion-inoculated and control mice was performed essentially as described previously [17,18].

### Antibody generation

Mouse monoclonal antibodies F010 and C067 against cystatin F were generated using mammalian cell display as previously described [19] with modifications. BALB/c mice were weekly immunized with his-tagged human recombinant cystatin F conjugated to Qβ virus-like particle (VPLs) [19] in alum four times. Splenocytes from immunized mice were harvested and stained with: FITC-labelled anti-mouse IgM/D, CD3, CD11c/b, CD4 and Gr-1; Qβ-bound human cystatin F, anti-Qβ serum (rabbit) and PE labelled anti-rabbit IgG; PE-TexasRed labelled anti-mouse CD19; Alexa 647 nm-labelled Qβ-VPLs (Cytos Biotechnology). Cystatin F-specific B cells (cystatin F/CD19 positive, Qβ-VPLs/IgM-/IgD-/CD3-/CD11c/b-/CD4-/Gr-1-negative) were sorted on a FACS Vantage SE flow cytometer (Becton Dickinson). Total RNA was isolated from Cystatin F-specific B cells and used to generate a Sindbis virus-based scFv library [19,20]. BHK cells were infected with the Sindbis virus-based single chain fragment variable (scFv) library and stained with Qβ-conjugated human cystatin F, anti-Qβ serum (rabbit) and PE labelled anti-rabbit IgG and a rabbit anti-sindbis virus serum and Cy5-labelled anti-rabbit IgG (Cytos Biotechnology), in the presence of propidium iodide. Infected BHK cells displaying cystatin F-specific scFv were individually sorted on 24-well plates containing BHK feeder cells. At least 48 h after sorting/plating, cells from wells with signs of infection were tested by flow cytometry for expression of cystatin F-binding scFv to identify viral clones encoding cystatin F-specific scFv. Fusion single chain fragment variable/fragment crystallizable (scFv-Fc) proteins were generated as previously described [19] and tested for binding to human recombinant cystatin F (Cytos Biotechnology) in ELISA assays using standard procedures. For expression as mouse IgG2aκ monoclonal antibodies, the heavy chain variable region (HCVR) and light chain variable region (LCVR) coding segments of the cystatin F-binding scFv molecules were PCR amplified using the following primer pairs (5’→3’, Microsynth): HCVR forward: CGA GGT GCA GCT GCT CGA GTC TGG GGC TGA GCT; HVCR reverse; GAC AGA TGG GCC CGT TGT TTT GGC TGA GGA GAC; LCVR forward: GAT ATT GAG CTC ACC CAG TCT CAA AAA TTC ATG; LCVR reverse: GCC ACC AGA GGA TTT GAT ATC CAG CTT GGT CCC.

Heavy chain and light chain encoding regions were cloned into a Epstein Barr Virus-based, pCB15 episomal expression vector allowing co-expression of immunoglobulin heavy and light chains under the cytomegalovirus promoter [19]. Expression vectors were transfected into HEK293T cells using Lipofectamin Plus (Invitrogen) and antibodies were purified from cell supernatants using protein A-Sepharose columns (GE healthcare).

### Tissue processing for Western blotting and ELISA

Human brain tissues from the NRPE-autopsy bio-bank were snap frozen and stored at -80°C. Ten-twenty percent brain homogenates (w/vol) were prepared in 2% Sacrosyl (Sigma) and PBS, using lysing matrix tubes (MP Bio) and a Precellys 24 (Bertin Technologies). Samples were spun down to remove gross debris and supernatants were stored at -80° C until further analysis.

Mice were deeply anesthetized with ketamine‐xylazine, transcardially perfused with a solution of PBS containing heparin and dissected organs were snap frozen and kept at -80°C until further analysis. Ten percent (w/vol) homogenates were prepared in either RIPA buffer (25mM Tris-HCl pH 7.6, 150mM NaCl, 1% NP-40, 1% sodium deoxycholate, 0.1% SDS) + Complete Mini Protease Inhibitor Cocktail (Roche) or in PBS + sucrose 0.32 M (Sigma), using Stainless Steel Beads 5 mm (Qiagen) and Tissue Lyser LT (Qiagen). Samples were spun down to remove gross debris and supernatants were stored at -80° C until further analysis.

### ELISA

Sandwich ELISA was performed on 96-well ELISA plates (F96 Cert. Maxisorp Nunc-immuno plate) coated with 5 μg/mL of the monoclonal mouse anti-cystatin F antibody F010 in PBS (pH = 7.2, Gibco). After overnight incubation, plates were washed 5 times with PBS supplemented with 0.1% (vol/vol) Tween-20 (PBST) using a 96-well plate washer (BioTek Instruments, ELx405). Nonspecific binding sites were subsequently blocked with 5% (w/vol) fat-free milk in PBST for 2h at room temperature. Recombinant cystatin F (AJ Roboscreen) was subjected to 1:2 serial dilution in sample diluent buffer (1% w/vol fat-free milk in PBST) to generate a standard curve with the following concentrations: 1000, 500, 250, 125, 62.5, 31.25, 15.6, 7.8 pg/ml. Blocking buffer was removed and standards/samples were loaded in either duplicates (undiluted CSF) or triplicates (cell lysates and tissue homogenates, diluted in sample diluent buffer). Plates were incubated for 2h at 37°C and washed 5 times. For detection of the captured antigen, plates were incubated with 0.75 μg/ml of the biotinylated monoclonal mouse anti-cystatin F antibody C067 for 1h at 37°C, then washed 5 times. Avidin-horseradish-peroxidase HRP (BD Pharmingen) diluted 1:1000 in sample diluent was loaded. After incubation of 45 min at 37°C plates were washed 5 times and developed with 3,3’,5,5’-tetramethylbenzidine substrate solution (high sensitivity TMB, BioLegend), for 10 min at room temperature in the dark. The reaction was stopped by adding stop solution, 0.18 M H_2_SO_4_. Plates were read at wavelength of 450 nm on a Versamax SNB plate reader (Molecular Devices).

Standard curves were generated using a four-parameter logistic (4PL) equation to calculate concentrations of unknown samples. Values of all 4 parameters were determined by SOFTmax PRO software (Molecular Devices).

To assess matrix effects in the assay, recombinant human cystatin F (Cytos Biotechnology) was spiked in pooled human CSF with the concentrations 1000, 500, 250, 125, 62.5, 31.25, 15.6 pg/ml and compared to the observed values with the expected protein levels diluted only in sample diluent buffer. We obtained measured levels differing up to 22% with respect to nominal values in the range between 62.5 and 1000 pg/mL. Below 62.5 the discrepancy was larger, possibly due to the presence of endogenous cystatin F in pooled human CSF used for spiking. Macroscopically haemorrhagic CSF samples were excluded, as preliminary results indicated extensively elevated cystatin F concentrations due to blood contamination.

Intra-assay variability was evaluated by spiking recombinant cystatin F into pooled human CSF at the following concentrations: 500, 62.5, 7.8 pg/ml. Validation samples were measured in 10 replicates. The coefficient of variation (%CV) was 2.5%, 6.2% and 10.5%, respectively.

To account for possible inter-assay variability, for measurements in mouse brain homogenates from different experiments, cystatin F levels are normalized to levels in NBH-injected control mice, whereas for measurements in human CSF samples 3 pre-defined CSF cases were employed as internal calibrators to pool data for the evaluation of cystatin F levels in CSF samples from cohort 2.

### Western blotting

Total protein concentration of tissue homogenates was measured using the BCA Protein Assay (Pierce), according to the manufacturer’s instructions. For proteinase K (PK) digestion, PK (100 μg/ml, pH = 7, Roche) was added aiming for a final concentration of 25 μg/ml and incubated at 37°C for 30 min; the reaction was stopped by adding NuPAGE 4xLDS sample buffer (Invitrogen) with β-mercaptoethanol (Sigma-Aldrich) and boiling at 95°C for 10 min. Separation was performed on a NuPAGE 12% Bis-Tris gel (Invitrogen) and transferred onto a Protran Nitrocellulose Transfer Membrane (Whatman) using the NuPAGE Gel Electrophoresis System (Invitrogen) or the Mini Trans-Blot cell System (Bio-Rad). After blotting, the membrane was washed once with PBS supplemented with 0.1% (vol/vol) Tween-20 (PBST), blocked with 5% (w/vol) fat-free milk in PBST and decorated with mouse anti-PrP POM1 [21] (300 ng/ml in 1% (w/vol) fat-free milk in PBST) overnight at 4°C. After washing, the membrane was probed with rabbit anti-mouse IgG2a antibody (1:10’000 dilution, Zymed). Blots were developed using Luminata Western HRP Substrates (Millipore) and visualized using either a Stella (Raytest) or LAS-3000 (Fujifilm) luminescent image analyzer. After initial image acquisition, membranes were washed, reprobed with mouse anti-actin monoclonal antibody (1:10’000 dilution, Millipore) and the same procedure was followed for acquisition of the actin signal. Uniform adjustment of contrast and levels on the acquired images and subsequent cropping were performed using Adobe Photoshop. Unprocessed Western blot images are provided in S1 File.

### Immunohistochemistry

Tissues were fixed in formalin, treated with concentrated formic acid to inactivate prions and embedded in paraffin. Tissue sections were subjected to deparaffinization through graded alcohols and heat-induced antigen retrieval in EDTA-based buffers. Stainings were performed on a NEXES immunohistochemistry robot (Ventana instruments) or on a BOND-III robot (Leica) using the following antibodies: 1:200 dilution of 3F4 (stock concentration 2 mg/ml, purified in house) for PrP staining; 1:3000 dilution of 4G8 (Signet) for Aβ staining; 1:1000 dilution of AT8 (Thermo Fisher Scientific) for staining of phosphorylated tau; 1:200 dilution of HPA040442 (Sigma) for cystatin F; 1:1000 dilution of rabbit anti-Iba1 (Wako). Immunoreactivity was visualized using an IVIEW DAB Detection Kit (Ventana) or Bond Polymer Refine Detection Kit (Leica). Haematoxylin and eosin staining was performed according to a standard protocol. For image analysis and acquisition, slides were scanned with NanoZoomer and images were obtained using the NanoZoomer Digital Pathology System (NDPview, Hamamatsu Photonics).

### Statistical analysis

Microarray data from a previous study [6] were re-analyzed using the perfect match-mismatch model-based expression analysis algorithm (www.dchip.org). Differentially expressed genes were defined as having an absolute fold change ≥1.3, a confidence interval >90% and Student’s t test p value <0.05 when comparing expression values between RML-infected vs. NBH-injected control mice.

Statistical analysis was performed using Graphpad Prism software and SPSS. Significance between two groups was determined by unpaired Student’s *t*-test. Comparison between multiple groups was assessed by One-way ANOVA followed by Bonferroni’s or Dunnet’s Multiple Comparison Test or Fisher’s least significant difference (LSD) *post-hoc* test, as stated in corresponding figure legends. P-values <0.05 were considered as statistically significant. For the statistical analysis, a normalizing log-transformation of murine *Cst7* mRNA levels and of mouse and human cystatin F values was applied. A multi-factorial analysis of co-variance (ANCOVA) of the log-transformed analysis variable cystatin F was performed on the data from Cohort 2 using SPSS. Categorical ANCOVA factors were diagnosis group and sex; continuous covariates were age of patient, age of CSF sample and total protein concentration. An additional term was included in the ANCOVA model to account for a possible interaction between sex and diagnosis.

## Results

### Microarray analysis of prion-infected mouse brains unveils *Cst7* as a candidate biomarker

We had previously performed a genome-wide, microarray-based transcriptomic analysis on brains from C57BL/6J mice at 145 days after intraperitoneal injection with RML prions (or non-infectious brain homogenate, NBH, as control) [6]. Under this experimental paradigm, the onset of clinical signs occurs at around 180 dpi, whereas the terminal stage is achieved at around 200 dpi in prion-inoculated mice. Microarray data were initially analyzed using the perfect-match-only model-based expression analysis algorithm, which relies on hybridization intensities of the perfect match probes, 25mers containing the canonical sequence for the corresponding transcripts. This approach allowed the identification of 77 differentially expressed transcripts, including *Serpina3n* [6].

**Fig 1:**
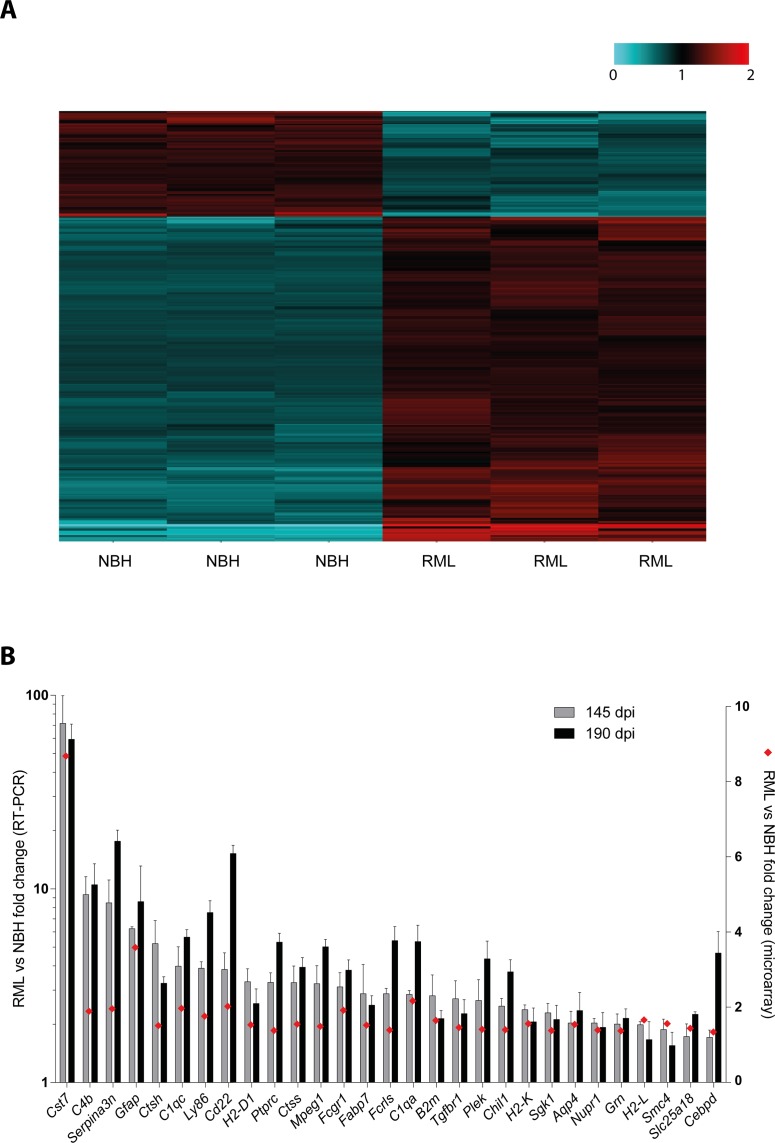
Microarray analysis identifies Cst7 as the most upregulated transcript in RML inoculated mice. **A** Heatmap showing relative expression of 251 differentially expressed genes (absolute fold change ≥1.3, a confidence interval >90% and Student’s t test p value <0.05) between RML prion-inoculated mice (RML, n = 3, A to C) and control mice injected with non-infectious brain homogenates (NBH, n = 3, A to C). For each gene, relative expression is defined as the ratio between the actual gene expression of each mouse and the average gene expression from all 6 analyzed mice (the latter set as 1, black) and is reported as grades of blue (downregulation) or red (upregulation). Expression data were obtained through the employment of the perfect match mismatch model-based expression analysis algorithm of microarray data previously reported in [6]. The list of differentially expressed genes is reported in S4 Table. **B** Validation of the upregulation of 29 transcripts (including *Cst7*) in RML-inoculated mice (n = 3) compared to NBH-injected controls (n = 3). Bars (left y axis) depict mean and standard deviation of transcript fold change expression between brains of RML-inoculated mice and NBH-injected controls (after normalization to *Actb* levels) at 145 and 190 days post-inoculation (dpi), as assessed by RT-PCR. Red diamonds (right y axis) depict transcript fold change expression between brains of RML-inoculated mice and NBH-injected controls at 145 dpi as assessed by microarray.

To identify additional transcripts differentially expressed in the early, pre-clinical phases of experimental prion disease, we have re-analyzed this dataset. We have taken advantage of the presence, within the employed microarray chip, of the mismatch probes. These are 25mer probes containing a deliberate mutation at position 13 and as such designed to measure non-specific or cross-hybridization [22,23]. We have therefore applied the perfect-match mismatch model-based expression analysis algorithm and focused on differentially expressed genes with absolute fold change ≥1.3, a confidence interval >90% and Student’s t test p value <0.05. By applying these filters, we identified 278 differentially expressed probes, corresponding to 259 genes, of which 201 genes were upregulated and 58 genes were downregulated in the brain of prion-infected vs. control mice (Fig 1A, S4 Table). Of note, the majority (≈78%) of these differentially expressed probes were not previously identified when using the perfect-match only algorithm. Remarkably, among the newly identified differentially expressed genes was *Cst7*, which represented the gene undergoing the strongest modulation in brains of prion-infected mice, with an upregulation of ≈8.7 fold.

*Cst7* encodes for cystatin F, a secreted type-II cysteine protease inhibitor mainly expressed in immune cells [24–28]. The early and strong upregulation of *Cst7* mRNA during prion disease, along with the fact that cystatin F can be secreted into the extra-cellular space, prompted us to investigate the role of cystatin F as a possible surrogate biomarker in prion diseases.

### Levels of *Cst7* transcripts during prion pathogenesis

To validate the upregulation of *Cst7* transcripts during prion disease identified with the microarray, we performed quantitative RT-PCR analysis in brains of prion-infected versus control mice (the latter injected with NBH). We analyzed samples at 145 dpi, the same pre-symptomatic stage used for microarray analysis [6], and at 190 dpi, when prion-inoculated mice showed signs of disease and were close to the terminal stage. We measured levels of *Cst7*, as well as levels of 28 additional transcripts which were found, to different extent, as upregulated in prion-infected mice by microarray analysis. Quantitative RT-PCR analysis confirmed the upregulation of all investigated transcripts at both time points (Fig 1B). Compared to microarray analysis, RT-PCR showed a higher magnitude of upregulation, which, in the case of *Cst7*, was >40 fold in brains of prion-infected versus control mice (Fig 1A and 1B).

**Fig 2:**
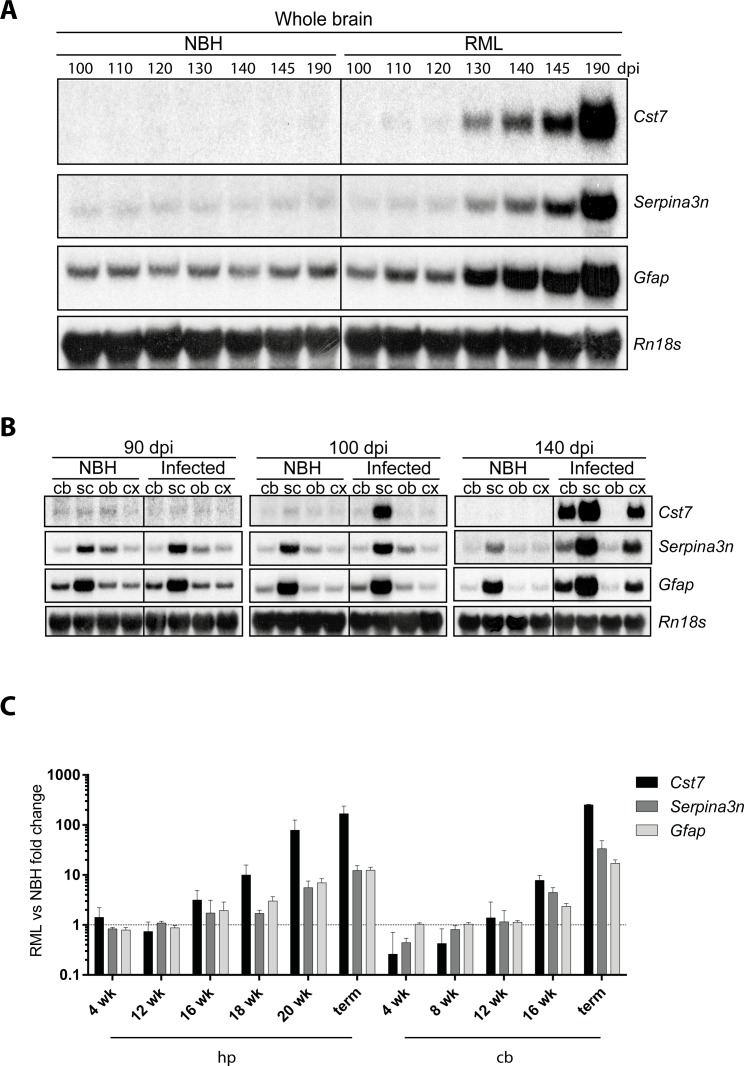
Spatial and temporal pattern of Cst7 upregulation in the central nervous system during experimental prion pathogenesis. **A** Northern hybridization analysis of *Cst7* mRNA in whole brains of mice at different time points (from 100 to 190 days post-inoculation, dpi) after injection with either non-infectious brain homogenate (NBH) or RML prions. For comparison, expression levels of *Serpina3n* and *Gfap* mRNA are also shown. Hybridization for *Rn18s*, encoding 18S rRNA, is performed for normalization. Each lane denotes a pool of brain extracts from 3 mice. **B** Northern hybridization analysis of *Cst7* mRNA in selected areas of the central nervous system (cb: cerebellum; sc: spinal cord; ob: olfactory bulb; cx: cortex) at different time points after injection with either NBH or RML prions. For comparison, expression levels of *Serpina3n* and *Gfap* mRNA are also shown. Hybridization for *Rn18s*, encoding 18S rRNA, is performed for normalization. Each lane denotes a pool of brain extracts from 3 mice. **C** *Cst7*, *Serpina3n* and *Gfap* mRNA levels in hippocampus (hp) and cerebellum (cb) of RML-inoculated mice (n = 3) compared to NBH-injected controls (n = 3). Bars depict mean and standard deviation of transcript fold change expression between brains of RML-inoculated mice and NBH-injected controls at different time points post-inoculation (expressed as week, wk), or at the terminal stage (term), as assessed RNA-sequencing. Dashed line indicates equal levels between RML-inoculated mice and NBH-injected controls (fold change of 1). Data concerning *Serpina3n*, *Gfap*, *Rn18s* and PK-resistant PrP from panels A and B reproduced, in modified form [6].

We then investigated the temporal pattern of *Cst7* transcription in the CNS after prion infection, and its relationship to the accumulation of partially protease-K resistant PrP. Northern blotting indicated an initial upregulation of *Cst7* transcripts already at 110 dpi (Fig 2A), when PrP^Sc^ becomes detectable by Western blotting in whole brains of prion-infected mice (S1 Fig). The PrP^Sc^ signal progressively increased over time (Fig 2A). Compared to *Serpina3n* and to *Gfap*, a marker of astrogliosis typically associated to prion disease, *Cst7* mRNA upregulation occurred earlier during prion disease (Fig 2A).

We next analyzed the course of *Cst7* upregulation in selected areas of the CNS. *Cst7* transcripts were found to be upregulated in prion-infected spinal cords at 100 dpi (Fig 2B). By 140 dpi *Cst7* mRNA levels were further increased in spinal cord and *Cst7* upregulation became evident also in cerebellum and in cerebral cortex (Fig 2B). Again, *Cst7* mRNA upregulation occurred earlier with respect to *Serpina3n* and *Gfap* mRNA levels (Fig 2B).

We then used RNA sequencing to assess the number of *Cst7* mRNA transcripts in the hippocampi and cerebella of mice at various time points after intracerebral injection with either RML prions or NBH (as control). In the hippocampus, *Cst7* showed a trend toward upregulation at 110 and 123 dpi (≈3 fold and 10 fold, respectively), which reached statistical significance at 137 dpi (≈79 fold, p<0.05, One-way ANOVA, Dunnett’s Multiple Comparison Test) and at the terminal stage of disease (≈168 fold, p<0.001, One-way ANOVA, Dunnett’s Multiple Comparison Test; Fig 2C, S5 Table). At these two time points, the increase of *Cst7* transcripts was significantly larger than that of *Serpina3n* and *Gfap* (for both time points p<0.0001, Two-way ANOVA, Bonferroni’s Multiple Comparison Test).

In the cerebellum, *Cst7* was significantly upregulated at 110 dpi (≈8 fold, p<0.05, One-way ANOVA, Dunnett’s Multiple Comparison Test), as well as at the terminal stage (≈251 fold, p<0.001, One-way ANOVA, Dunnett’s Multiple Comparison Test) compared to NBH-injected mice (Fig 2C, S5 Table). At the terminal stage, *Cst7* upregulation was significantly higher than that of *Serpina3n* and *Gfap* (p<0.0001, Two-way ANOVA, Bonferroni’s Multiple Comparison Test). In summary, *Cst7* mRNA upregulation occurred earlier and was larger than that of *Serpina3n* and *Gfap* (Fig 2C). Collectively, these data indicate that in C57BL/6 mice intraperitoneally or intracerebrally inoculated with RML prions, early, progressive and robust upregulation of *Cst7* mRNA occurs throughout the disease course.

### Levels of *Cst7* mRNA in other neurologic conditions

To study the cellular pattern of *Cst7* expression within the mouse brain, we interrogated the RNA-sequencing-based transcriptome database for mouse brain cortex cells [14]. This analysis revealed that *Cst7* ranked at the 48^th^ percentile of expression of all mouse genes and that *Cst7* transcripts were significantly enriched in microglia/macrophages as compared to other cell types of the brain cortex (S2 Fig), in line with the notion that *Cst7*/Cystatin F is mainly expressed in immune cells, including microglia [29,30]. Of interest, transcriptomic analysis of different cell types of the human brain [15] showed that *CST7* ranked t the 43^rd^ percentile of expression of all human genes and that *CST7* transcripts are mainly detected in microglia/macrophages and endothelial cells (S2 Fig). Next, we analysed *Cst7* mRNA levels in mouse microglial cells during development and after an immune challenge with lipopolysaccharides using the RNA sequencing-based transcriptome database for mouse microglia cells[16]. This showed that *Cst7* mRNA levels are stable during microglia maturation, whereas they show a trend toward increase upon exposure to lipopolysaccharides (S2 Fig).

In light of these data, we asked whether neurologic conditions with prominent neuroinflammation/microgliosis other than prion disease may result in the upregulation of *Cst7* mRNA. *Cst7* mRNA overexpression as assessed by Northern blotting was evident to different extents in the brains of terminally sick ΔF mice expressing a toxic, truncated PrP^C^ molecule [31], in mice with experimental autoimmune encephalitis [11], a model of multiple sclerosis, and in aged TgAPP23 mice overexpressing human APP^Swe/V717I^ [32], a model for Alzheimer’s disease (AD) (S2 Fig). This analysis showed that mild brain *Cst7* overexpression occurs in other mouse models of neurologic conditions with neuroinflammation/microgliosis.

### Cystatin F protein levels during experimental prion pathogenesis

We next aimed at studying cystatin F protein levels in mouse models of prion diseases. In naïve C57BL/6 mice, cystatin F levels oin spinal cord, cerebellum and forebrain were significantly lower than in thymus, in line with RT-PCR data of *Cst7* mRNA in the same organs (S3 Fig).

**Fig 3:**
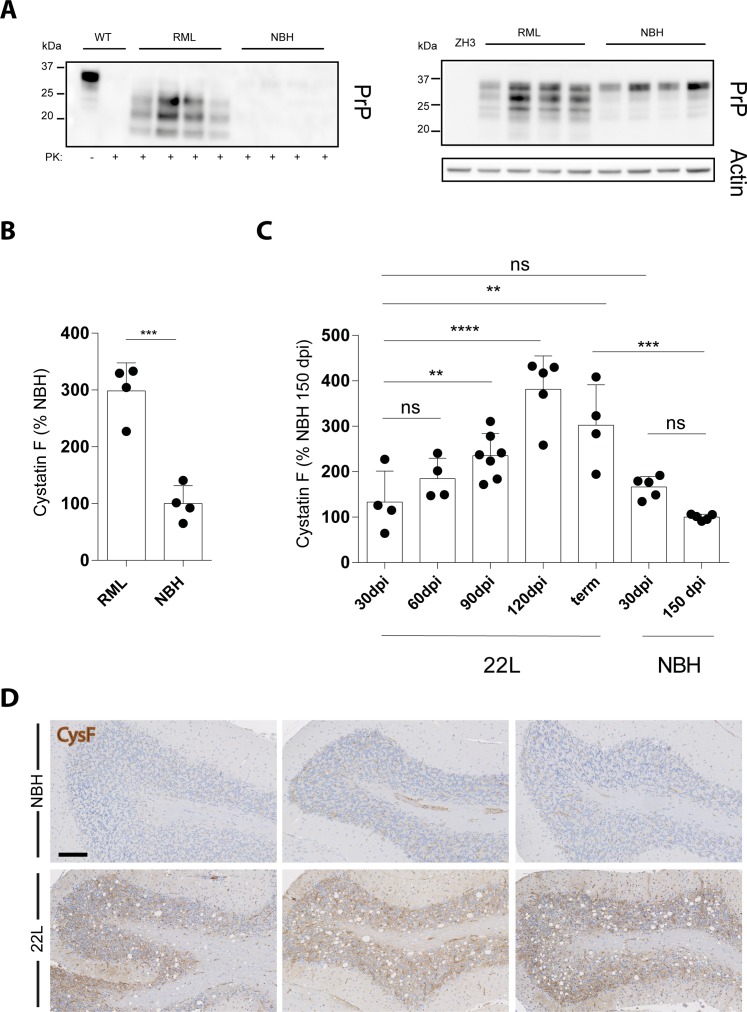
*Cst7*/cystatin F brain expression during prion pathogenesis. **A** Western blotting analysis in brain extracts of terminally sick, prion-infected mice (RML), and of mice injected with non-infectious brain homogenate (NBH), showing the amount of partially protease K (PK)-resistant prion protein (PrP) (left panel). Identical protein extracts omitting proteinase K treatment were used for Western blotting with an anti-PrP and anti-actin antibody to verify equivalent loading in each lane (right panel). ZH3 denotes brain from a C57BL/6J-*Prnp*^ZH3/ZH3^ mouse as control. Each lane denotes one mouse. **B** Cystatin F levels in brain extracts from the same mice depicted in A, relative to levels in NBH-injected mice (set as 100%; ***, p<0.001, Student’s t test). **C** Cystatin F levels in brain extracts from mice injected with either non-infectious brain homogenate (NBH) or 22L prions at different time points (dpi, days post-inoculation; term, terminal prion disease, reached at approximately 150 pdi in this experiment), relative to levels in NBH-injected mice at 150 dpi (ns, p>0.05; **, p<0.01; ***, p<0.001; ****, p<0.0001, one-way ANOVA followed by Bonferroni’s Multiple Comparison test). **D** Histological analysis of cystatin F expression in cerebella of 22L-inoculated mice at the terminal stage of the disease and the relative NBH-injected controls. Three representative mice per each group are depicted. Scale bar: 100 μm.

However, compared to NBH-injected control mice, RML prion-inoculated, terminally-sick mice showed significantly higher levels of brain cystatin F (Fig 3A and 3B). We also studied cystatin F levels in brains of C57BL/6 mice at different time points after intracerebral challenge with the 22L inoculum, another strain of rodent-adapted scrapie prions. Compared to NBH-injected control mice, a trend toward increased cystatin F levels was evident in brains of 22L-inoculated mice already at 60 dpi, and at 90 dpi this difference reached statistical significance and was maintained throughout the disease course, until the terminal stage, which was reached at around 150 dpi (Fig 3C). Also, immunohistochemical analysis showed an increase in cystatin F protein expression in brains of terminally-sick, 22L-inoculated mice compared with NBH-injected controls, with a diffuse pattern compatible with the fact that the protein can be released in the extracellular space (Fig 3D).

### Cystatin F protein levels in human Cerebrospinal Fluid (CSF) samples

To gain a first insight into cystatin F levels in neurologic conditions other than prion diseases, we analyzed a first cohort of patients with several neurodegenerative and neuro-infectious/inflammatory conditions. This cohort included patients with various forms of cognitive impairment (mild cognitive impairment, late-onset Alzheimer’s disease and fronto-temporal dementia n = 4), Parkinson’s disease (n = 2), bacterial and viral meningitis (n = 3 and 8, respectively), limbic encephalitis (n = 3), multiple sclerosis (n = 6) and neuroborreliosis (n = 3). Cystatin F was detected in all CSF samples, with the highest levels observed in samples from the bacterial meningitis groups (S4 Fig, p<0.01 compared to dementia and to multiple sclerosis, p<0.05 compared to encephalitis, one-way ANOVA followed by Bonferroni’s *post-hoc* test).

We next explored the role of cystatin F as a potential biomarker for prion diseases. For this purpose, we analyzed CSF samples from patients referred to the NRPE for 14-3-3 analysis because of clinical suspicion of prion disease. Of these cases, we included only cases of autopsy-confirmed Creutzfeldt-Jakob disease or Alzheimer’s disease, whereas non-prion related diagnoses were excluded. Cases evaluated at the Neurology Department of the University of Zurich with available CSF samples and with a definitive autopsy-based diagnosis were also included. This resulted in 11 cases of definitive Alzheimer’s disease (“Def AD”) and 39 cases of definitive CJD (“Def CJD”). In addition, we also included 26 cases of Alzheimer’s disease diagnosed clinically based on stringent criteria (“Clin AD”), which were matched to the Def AD and Def CJD groups by patient age as well as gender, and sample age. We included 24 cases with no neurodegenerative condition (“Controls”), consisting of subjects undergoing lumbar puncture during the diagnostic work-up for headache, vertigo, or sleep disorders. This latter group was matched to the others based on the sample age.

**Fig 4:**
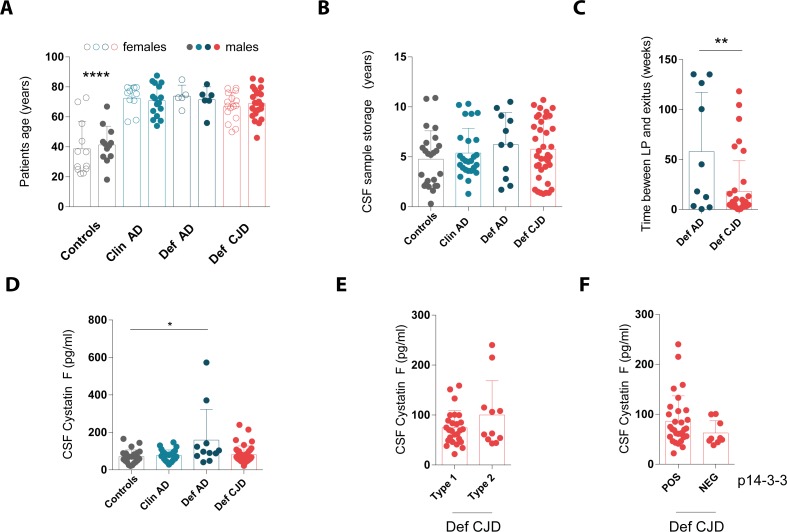
Cystatin F levels in cerebrospinal fluid from patients with Creutzfeldt-Jakob diseases, Alzheimer’s disease and controls. Cystatin F levels were measured in patients with autopsy-confirmed definitive Creutzfeldt-Jakob disease (Def CJD) or Alzheimer’s disease (Def AD), clinically diagnosed Alzheimer’s disease (Clin AD) and non-demented controls (Controls). **A** Age and gender distribution (****, p<0.0001 against all other categories, one-way ANOVA followed by Bonferroni’s Multiple Comparison test). **B** Sample storage of archival cerebrospinal fluid (CSF). **C** Time interval between lumbar puncture (LP) and *exitus* for deceased patients with autopsy-confirmed diagnosis of neurodegeneration (Def AD and Def CJD; **, p<0.01, Student’s t test). **D** Cystatin F levels in CSF from all study subjects from cohort 2 (*, p<0.05, one-way ANOVA followed by Bonferroni’s Multiple Comparison test). **E** Sub-analysis of cystatin F levels in CSF from Def CJD subjects according to the PrP type[35]. **F** Sub-analysis of cystatin F levels in CSF from Def CJD subjects according to the results of p14-3-3 Western blotting analysis as performed at the time of CSF sample collection. In all panels: Dots: denote individual subjects; bars: mean; error bars: standard deviation.

No significant differences were observed in patient’s age and gender distribution as well as CSF sample storage time among Def CJD, Def AD and, as a result of the matching process, Clin AD (Fig 4A and 4B). Instead, subjects from the control group were significantly younger compared to the other groups but also in this case CSF sample storage time was similar thanks to the matching process (Fig 4A and 4B). For cases for which an autopsy was performed (namely Def AD and Def CJD groups), the time between lumbar puncture and the *exitus* was significantly shorter for the Def CJD group (p = 0.0051, Student’s t test), in line with the more rapid clinical course [33,34].

The highest cystatin F CSF levels were found in the Def AD group and these values were significantly different compared to levels in the Controls (p<0.05, one-way ANOVA followed by Bonferroni’s Multiple Comparison test). Notably, no significant difference was seen between Def CJD and Controls (p = 1, one-way ANOVA), Def CJD and Def AD (p = 0.054, one-way ANOVA) and Def CJD and Clin AD (p = 0.3, one-way ANOVA). Analysis of covariance was performed on the log-transformed cystatin F levels taking into account possible influences of sex, patient’s age at the time of lumbar puncture and age of CSF sample. There was no association between diagnosis or gender and cystatin F levels (p = 0.239, ANCOVA). However, patient’s age was associated with cystatin F levels (p = 0.007, ANCOVA). Further analysis showed that only for the Def AD group cystatin F levels correlate with patient’s age (r = 0.667, p = 0.025).

Within the Def CJD group, there was no significant difference of cystatin F levels between PrP type 1 and type 2 cases [35] (p = 0.14, Student’s t test, Fig 4E), nor between patients with 14-3-3 positive and negative results (p = 0.19, Student’s t test, Fig 4F).

Collectively, these analyses indicate the lack of a significant increase of cystatin F levels in CSF samples of Creutzfeldt-Jakob disease cases compared to clinically relevant controls.

### Cystatin F protein levels in human brain samples

We next aimed at comparing cystatin F levels in the brain parenchyma of Creutzfeldt-Jakob disease vs. Alzheimer’s disease patients. To this purpose, we examined a third cohort comprising 11 and 8 autopsy-confirmed cases, respectively. We focused on frontal cortex, a brain area commonly affected both by amyloid β pathology in Alzheimer’s disease and by partially PK-resistant PrP in Creutzfeldt-Jakob disease, as well as on cerebellum, which is rarely involved by amyloid β pathology in Alzheimer’s disease [7,36,37].

**Fig 5:**
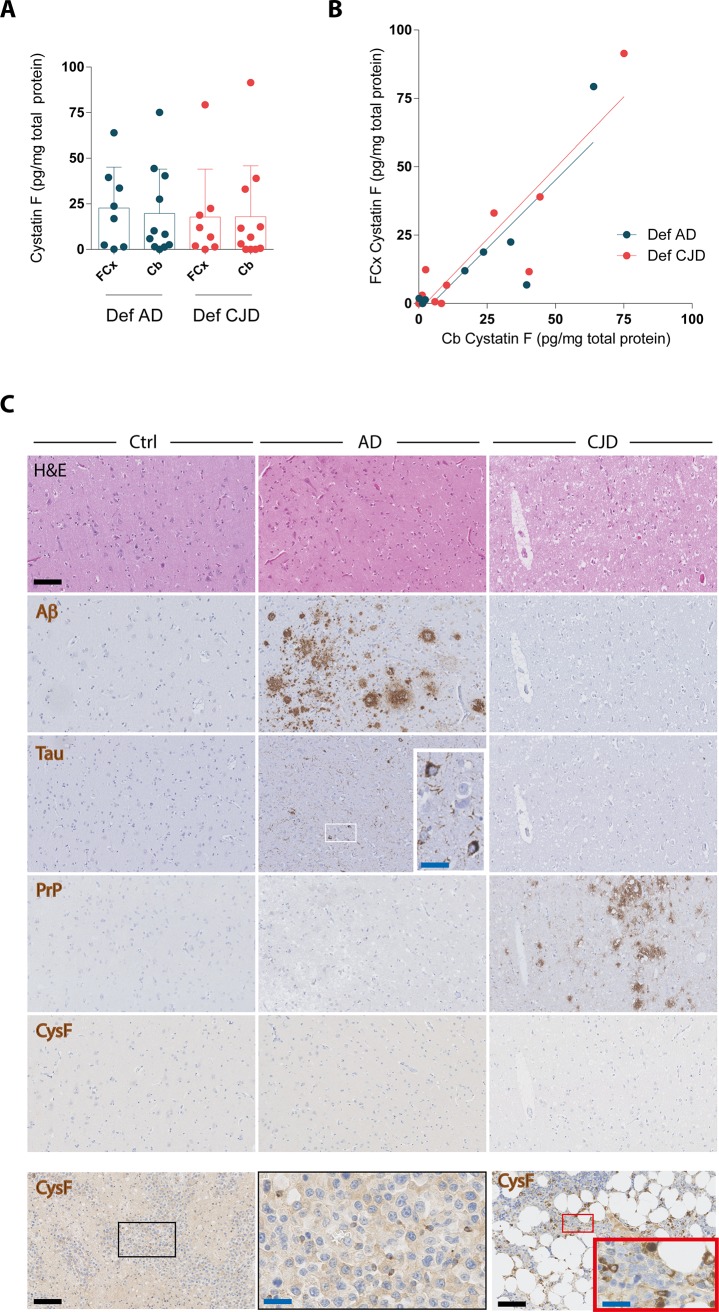
Cystatin F levels in brains from patients with Creutzfeldt-Jakob disease and Alzheimer’s disease. **A** Cystatin F levels in frontal cortex (FCx) and cerebellum (Cb) of patients with autopsy-confirmed definitive Creutzfeldt-Jakob disease (Def CJD) or Alzheimer’s disease (Def AD). Dots: denote individual subjects; bars: mean; error bars: standard deviation. **B** Correlation of cystatin F levels in the two regions of each patient, denoted by a dot (R^2^ 0.73 for Def AD and 0.83 for CJD, linear regression analysis). **C** Histologic analysis of cystatin F expression in frontal cortex of the same cohort of patients. Regions with abundant pathology, including amyloid β (Aβ) plaques and neurofibrillary tangles (Tau, further magnified in the inset) in Alzheimer’s disease and spongiosis and partially protease resistant prion protein (PrP) in Creutzfeldt-Jakob disease, from representative cases are depicted. Non-demented subjects were included as control (Ctrl). Lower row, left: encephalitis, with cystatin F positive infiltrating cells (further magnified in the inset at center). Lower row, right: bone marrow, with cystatin F positive cells (further magnified in the inset). Scale bar: 100 μm (black) or 25 μm (blue).

Cystatin F levels were not significantly different between patients with Creutzfeldt-Jakob disease and Alzheimer’s disease, both sampled from the frontal cortex and cerebellum (Fig 5A), with linear regression analysis indicating a correlation of cystatin F levels in the two regions of the same case (Fig 5B). Also, within each diagnostic group, no significant difference was observed between cystatin F levels in the frontal cortex and in the cerebellum (Fig 5A). Moreover, immunohistochemical analysis could not detect any cystatin F in the frontal cortex of these patients with Creutzfeldt-Jakob disease and Alzheimer’s disease, despite the presence of microgliosis as detected by Iba1 stainings (S5 Fig) and the presence of partially protease-resistant PrP in the former and amyloid β deposits and neurofibrillary tangles in the latter (Fig 5C). Conversely, cystatin F was detectable, mainly in infiltrating cells, in cases of encephalitis, as well as in other tissues known to express cystatin F such as bone marrow (Fig 5C). Collectively, these data indicate similar levels of cystatin F levels in brain parenchyma of patients with Creutzfeldt-Jakob disease and Alzheimer’s disease.

## Discussion and conclusion

A global transcriptomic analysis has identified *Cst7* as a highly upregulated transcript in the brain of prion-inoculated mice in the preclinical stage of the disease. Further analyses validated this finding, showing that *Cst7* upregulation occurs early during prion pathogenesis, parallels the appearance of partially PK-resistant PrP, and has a magnitude of change that is unmatched by other examined prion-induced transcripts. *Cst7* encodes cystatin F, which is mainly expressed by immune cells, with microglia being the main cellular source of *Cst7* expression within the brain. Importantly, upregulated *Cst7* translated into higher cystatin F protein levels in the brains of prion-inoculated mice. All these findings, together with the observation that cystatin F can be secreted in the extracellular space and is found in biological fluids, formed the rationale for investigating the possible role of cystatin F as a biomarker of prion diseases.

Microglial *Cst7* can be induced by lipopolysaccharide challenge, and moderately increased *Cst7* levels were observed in different mouse models of neurodegenerative and neuroinflammatory conditions with microgliosis. Therefore, we first analyzed a cohort of CSF samples from subjects with different neuro-infectious, -inflammatory and -degenerative diseases. Significantly increased levels of cystatin F were observed in the CSF of patients diagnosed with bacterial and viral meningitis. In light of the cellular origin of cystatin F, this observation possibly reflects blood-brain-barrier leakage and associated CSF leucocytosis that is often observed in the course of bacterial and viral meningitis [38,39]. The fact that infectious conditions of the central nervous system are accompanied by significantly increased CSF cystatin F levels does not necessarily represent a limitation, as in the vast majority of the cases these conditions are not relevant in the differential diagnosis of CJD.

We next measured cystatin F levels in CSF samples from patients with rapidly progressive dementia and autopsy-confirmed Creutzfeldt-Jakob or Alzheimer’s disease, as well as in patients with clinically diagnosed Alzheimer’s disease and non-demented controls. Unexpectedly, we found no significant increase in CSF levels of cystatin F among Creutzfeldt-Jakob disease cases compared to all other diagnostic groups.

A certain degree of pre-analytical variation, especially in sample withdrawal, processing, storage and shipping, may play a role as a confounding factor [40], since our work was a retrospective study mainly based on external CSF samples sent to our reference laboratory in the context of the clinical work-up for suspected Creutzfeldt-Jakob disease. On the other hand, we found no correlation between CSF sample age and cystatin F levels, and the measured total protein levels were in line with the different diagnoses (data not shown). This finding essentially excludes the likelihood of substantial protein degradation.

Metabolism, biodistribution and the half-life of cystatin F in different biological fluids, as well as the contribution of blood-borne vs brain-borne cystatin F within the CSF remain largely unknown. Taking these aspects into consideration, we analysed cystatin F levels in autoptic brain tissue to elucidate whether there was any significant upregulation of this protein in the brains of patients with Creutzfeldt-Jakob disease. Remarkably, no significant differences were observed between Creutzfeldt-Jakob disease and Alzheimer’s disease brain tissue, in line with the results in CSF samples.

Alternatively, brain-derived cystatin F may be preferentially retained or degraded within brain parenchyma, without significantly diffusing into the CSF. Cystatin C belongs to the cystatin type II super family–like cystatin F–and has amyloidogenic properties [41], and the Leu68Gln variant (*CST3* p.Leu94Gln) causes hereditary amyloidosis with cystatin C amyloid deposition within the CNS [42,43]. Further, *in vitro* and *in vivo* studies in mice indicate that cystatin C co-aggregates with amyloid β and thus inhibits oligomerization and fibril formation in Alzheimer’s disease models [44,45]. Similarly, cystatin F could be sequestered by PrP^Sc^ aggregates and accumulate in the brain instead of diffusing into the subarachnoid space and hence no elevated concentrations would be detectable in the CSF. To test for this hypothesis, we investigated cystatin F expression and distribution within brain tissue by immunohistochemistry. This analysis failed to detect a significant accumulation of cystatin F in brain areas with prominent PrP^Sc^ aggregates in Creutzfeldt-Jakob disease cases or amyloid β plaques in Alzheimer’s disease cases. In fact, cystatin F was barely detectable in patients with Creutzfeldt-Jakob disease and Alzheimer’s disease, similarly to non-demented controls, whereas cystatin F expression could be detected in inflammatory infiltrating cells in cases of meningitis.

It is also possible that prion-induced upregulation of *Cst7* is a prion strain-dependent phenomenon. This would not be unexpected, as prion strains transmit disease with distinctive phenotypes, which include neuropathological and biochemical changes [1,4]. For example, microglia activation, which plays a key neuroprotective role in prion diseases [46], was shown to vary considerably among different prion models [47]. However, substantial upregulation of *Cst7* transcripts was detected in cultured murine microglia infected with the Creutzfeldt-Jakob disease-derived Fukuoka strain [48], as well as in brains of mice infected with RML, ME7, 139A and 301V rodent-adapted scrapie prions [49–52]. Furthermore, we observed upregulation of both *Cst7* mRNA and cystatin F protein levels in brains of mice infected with RML and 22L prions.

These observations suggest that *Cst7* upregulation is remarkably conserved upon exposure to different prion strains, at least in mouse models of prion diseases. Whether *Cst7* upregulation occurs also in the context of field cases of animal prion disease has to be established. Of interest are the results of a recent study testing levels of selected transcripts in two CNS areas of a limited number of Creutzfeldt-Jakob disease cases, subtypes MM1 and VV2, and controls. Compared to controls, a trend towards slightly increased *CST7* levels was found in the frontal cortex of patients with Creutzfeldt-Jakob disease subtype MM1, and in the cerebellum of patients with Creutzfeldt-Jakob disease subtype VV2, yet none of these changes reached statistical significance [53]. Conversely, there was a significant upregulation of microglial transcripts *CD68*, *ITGAM* (encoding CD11b) and *Aif1* (encoding Iba1) in frontal cortex of MM1 cases and in cerebellum of VV2 cases [53]. This observation, together with the results of our studies, suggests that the presence of microgliosis is not sufficient to cause a significant increase in cystatin F levels in patients with Creutzfeldt-Jakob disease.

One possible explanation for the divergent observations in experimental and human prion disease may lie in the intrinsic differences between mouse and human immune system, which is shaped by a complex interplay of genetic, epigenetic and environmental factors, and exhibits significant differences in development, homeostasis and response to challenge [54]. Multiple divergent molecular pathways in immune cells have been described, including for macrophages [55] and microglia [56]. Also, transcriptional changes upon different pro-inflammatory stimuli significantly diverge between mice and humans [57], and differences in selected inflammatory molecules have been reported between brains of Creutzfeldt-Jakob disease patients and brains of transgenic mice overexpressing the human prion protein and inoculated with Creutzfeldt-Jakob disease-derived prions [53]. In this context, it is of interest to note that *Cst7* was among the most strongly upregulated transcripts in the hippocampus of young vs. old mice, ranking 2^nd^ and 12^th^ between 3 vs. 24 month-old mice and 3 vs. 29 month-old mice, respectively [58]. Conversely, its human homologue *CST7* was not differentially expressed in the aging human brain (The Human Brain Transcriptome atlas [59]). Also, *Cst7* transcripts are almost exclusively found in microglia/macrophages in mouse brain, whereas *CST7* transcripts are present both in microglia/macrophages and endothelial cells in the human brain [14,15].

The robust overexpression of *Cst7*/cystatin F in our investigated mouse models, together with the upregulation of *Serpina3n*/α1-anti-chimotrypsin in both mouse models and human cases of prion disease, suggests a role for cysteine protease inhibitors in prion pathogenesis. Cysteine proteases have been found to be involved in the clearance of partially PK-resistant PrP *in vitro* [60–62]. Also, upregulation of cysteine proteases has been reported in different cellular and animal models of prion diseases [47,50,63–65] and in brains of Creutzfeldt-Jakob disease patients [66]. Further studies may clarify whether changes in cysteine proteases, and inhibitors thereof, are directly involved in prion disease pathogenesis or instead represent reactive changes.

As cystatin F can be secreted in the extracellular space and is present in body fluids, we explored its potential as a biomarker of Creutzfeldt-Jakob disease, the prototypical prion disease in humans. However, we found no significant increase in cystatin F levels in either cerebrospinal fluid or brain parenchyma of patients with Creutzfeldt-Jakob disease compared to Alzheimer’s disease or non-demented controls. Our results demonstrate the existence of dramatic species differences between mice and humans in terms of brain cystatin F expression during prion disease. They also rule out cystatin F as a useful surrogate biomarker in Creutzfeldt-Jakob disease.

## Supporting information

S1 Fig Partially protease K-resistant PrP brain accumulation during prion disease. Western blotting analysis showing the amount of partially protease K (PK)-resistant prion protein (PrP) in whole-brain extracts of prion-infected mice at various dpi (upper membrane). Identical protein extracts omitting proteinase K treatment were used for western blotting with an anti-actin antibody to allow for normalization and to verify equivalent loading in each lane (lower membrane). Each lane denotes a brain extract from a representative mouse from each group.
(TIF)

S2 Fig *Cst7* brain expression in physiologic and pathologic conditions. **A** Expression levels of *Cst7* in different cell types of the mouse brain based on RNA sequencing profiling of acutely purified cell populations from the mouse brain cortex. FPKM: fragments per kilobase of transcript per million mapped reads. The oligodendrocyte precursor population is reported to have a 5% contamination with microglia based on whole transcriptome profile. Dots: denote individual cell preparations; bars: mean; error bars: standard deviation. Data are from the mouse brain transcriptome database [14]. **B** Expression levels of *CST7* in different cell types of the human brain based on RNA sequencing profiling of acutely purified cell populations from the human brain cortex. FPKM: fragments per kilobase of transcript per million mapped reads. Dots: denote individual cell preparations; bars: mean; error bars: standard deviation. Data are from the human brain transcriptome database [15]. **C** Expression levels of *Cst7* in different developmental stages of mouse microglia and in relation to the expression of the early microglial marker Tmem119 or to the treatment with lipopolysaccharide (LPS). En indicates embryonal day n; Pn indicates post-natal day n. Dots: denote individual cell preparations; bars: mean; error bars: standard deviation. Data are from the mouse developmental microglia dataset, Bennett et al. [16]. **C** Northern hybridization analysis of *Cst7* mRNA in whole brains of mice with different neurodegenerative/neuroinflammatory conditions, as compared with levels in brains of mice at 190 days post injection (dpi) with RML prions or non-infectious brain homogenate (NBH). ΔF: terminally sick mice expressing a toxic, truncated PrP^C^ molecule [31]; TgAPP23: aged mice overexpressing human APP^Swe/V717I^ [32]; EAE: mice with experimental autoimmune encephalitis induced by administration of MOG_35-55_ peptide emulsified in complete Freund’s adjuvant (CFA) and receiving an intraperitoneal injection with pertussis toxin [11]; CFA: EAE control mice were only the injection of the MOG_35-55_ peptide was omitted. Each lane denotes a pool of brain extracts from 3 mice.
(TIF)

S3 Fig *Cst7*/cystatin F expression in different organs in mice. Correlation between cystatin F protein levels (x axis) and *Cst7* mRNA levels (y axis) in thymus and different regions of the central nervous system (CNS) in adult C57BL/6J mice (n = 4 for all organs except n = 3 for spinal cord). Each dot denotes an individual organ or CNS area. Inset: magnification of the area delimited between the axis and the dashed lines.
(TIF)

S4 Fig Cystatin F levels in cerebrospinal fluid from patients with different neurologic conditions. Cystatin F levels were measured in patients with different neurological conditions (Bact. Meningitis, bacterial meningitis; Vir. meningitis, viral meningitis). Dots: denote individual subjects; bars: mean; error bars: standard deviation (*, p<0.05; **, p<0.01, one-way ANOVA followed by Bonferroni’s multiple comparison test).
(TIF)

S5 Fig Microglia in brains from analyzed patients with Creutzfeldt-Jakob disease and Alzheimer’s disease. Histologic analysis of microglia (IBA1) in frontal cortex of the same cohort of patients with Alzheimer’s disease (AD) and Creutzfeldt-Jakob disease (CJD) reported in Fig 5. Non-demented subjects were included as control (Ctrl). Images from two representative cases per group are depicted. Scale bar: 100 μm.
(TIF)

S1 File Uncropped and unmodified Western blot and Norther blot images. (PDF)

S1 Table Clinical assessment and scoring of wild-type mice inoculated with RML prions. (DOCX)

S2 Table Clinical assessment and scoring of wild-type mice after induction of experimental autoimmune encephalitis. (DOCX)

S3 Table List of primers used in this study. (DOCX)

S4 Table Differentially expressed probes as assessed by the perfect match-mismatch model-based analysis of microarray data. (XLSX)

S5 Table Expression data (as reads per kilobase per million mapped reads, RPKM) of *Cst7*, *Gfap* and *Serpina3n* during prion disease as assessed by RNA sequencing. (XLSX)
