# Supplementary figures and images for: Cystatin F is a biomarker of prion pathogenesis in mice
Source: PLoS One. 2017 Feb 8;12(2):e0171923. doi: 10.1371/journal.pone.0171923 (PMC5298286; doi:10.1371/journal.pone.0171923)

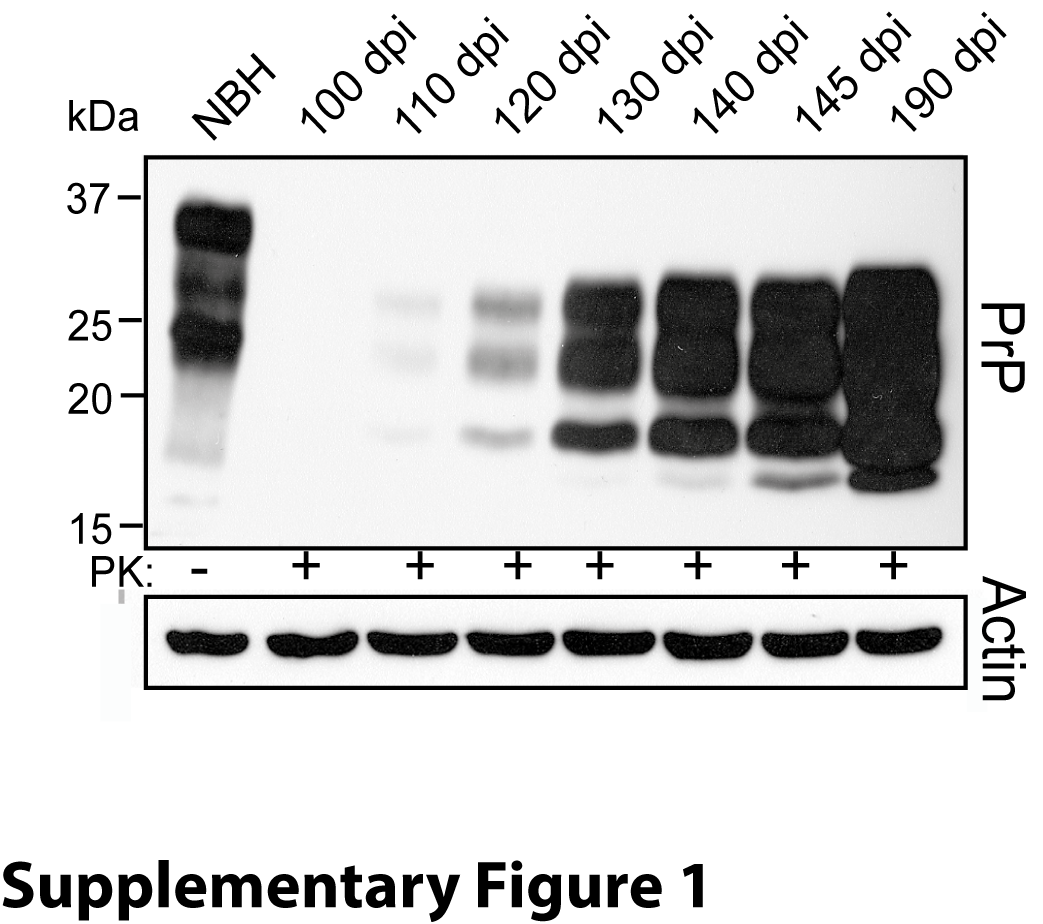

Supplement: S1 Fig — Western blotting analysis showing the amount of partially protease K (PK)-resistant prion protein (PrP) in whole-brain extracts of prion-infected mice at various dpi (upper membrane). Identical protein extracts omitting proteinase K treatment were used for western blotting with an anti-actin antibody to allow for normalization and to verify equivalent loading in each lane (lower membrane). Each lane denotes a brain extract from a representative mouse from each group. (TIF) [file pone.0171923.s001.tif]

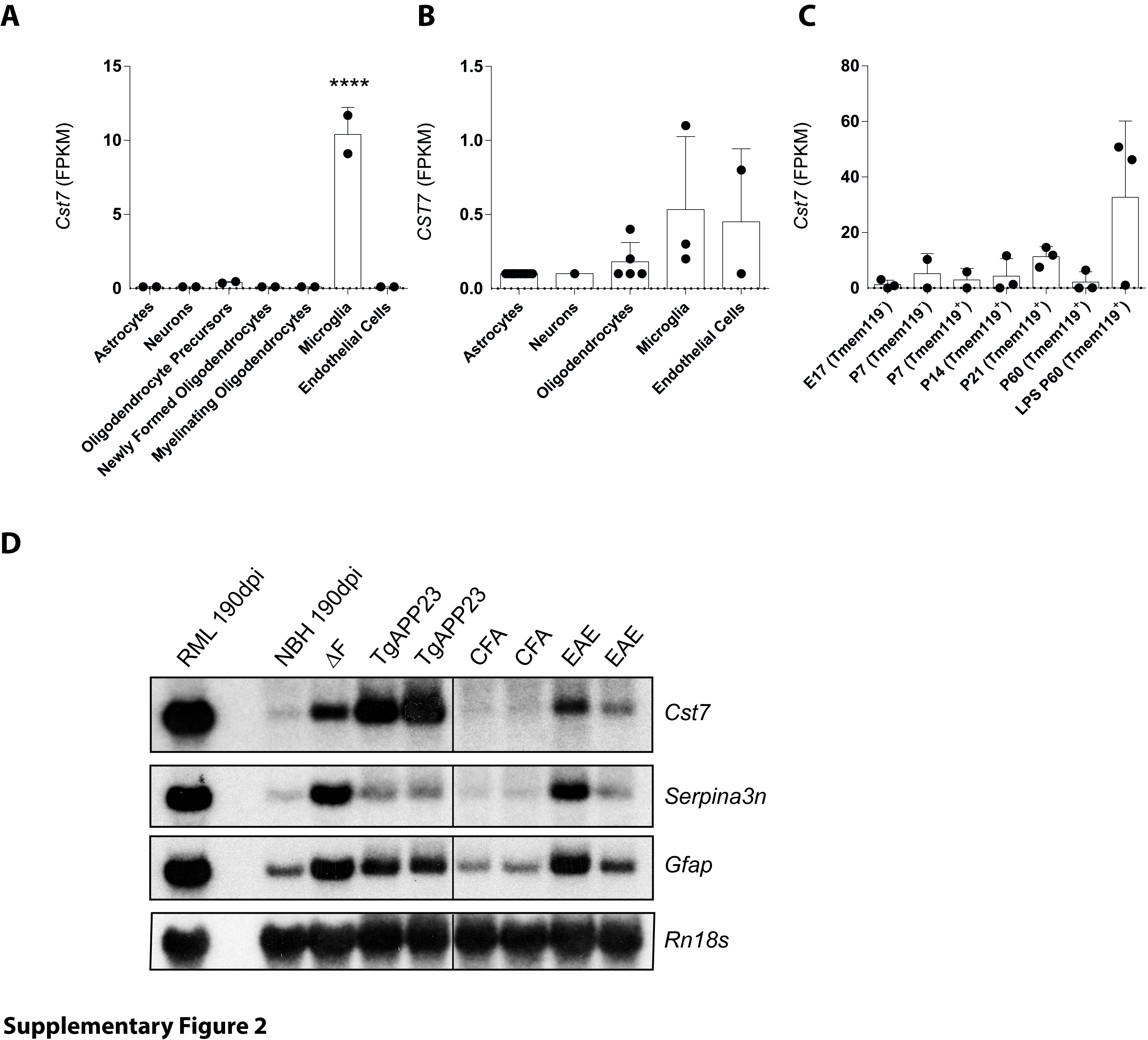

Supplement: S2 Fig — A Expression levels of Cst7 in different cell types of the mouse brain based on RNA sequencing profiling of acutely purified cell populations from the mouse brain cortex. FPKM: fragments per kilobase of transcript per million mapped reads. The oligodendrocyte precursor population is reported to have a 5% contamination with microglia based on whole transcriptome profile. Dots: denote individual cell preparations; bars: mean; error bars: standard deviation. Data are from the mouse brain transcriptome database [14]. B Expression levels of CST7 in different cell types of the human brain based on RNA sequencing profiling of acutely purified cell populations from the human brain cortex. FPKM: fragments per kilobase of transcript per million mapped reads. Dots: denote individual cell preparations; bars: mean; error bars: standard deviation. Data are from the human brain transcriptome database [15]. C Expression levels of Cst7 in different developmental stages of mouse microglia and in relation to the expression of the early microglial marker Tmem119 or to the treatment with lipopolysaccharide (LPS). En indicates embryonal day n; Pn indicates post-natal day n. Dots: denote individual cell preparations; bars: mean; error bars: standard deviation. Data are from the mouse developmental microglia dataset, Bennett et al. [16]. C Northern hybridization analysis of Cst7 mRNA in whole brains of mice with different neurodegenerative/neuroinflammatory conditions, as compared with levels in brains of mice at 190 days post injection (dpi) with RML prions or non-infectious brain homogenate (NBH). ΔF: terminally sick mice expressing a toxic, truncated PrPC molecule [31]; TgAPP23: aged mice overexpressing human APPSwe/V717I [32]; EAE: mice with experimental autoimmune encephalitis induced by administration of MOG35-55 peptide emulsified in complete Freund’s adjuvant (CFA) and receiving an intraperitoneal injection with pertussis toxin [11]; CFA: EAE control mice were only the injection of [file pone.0171923.s002.tif]

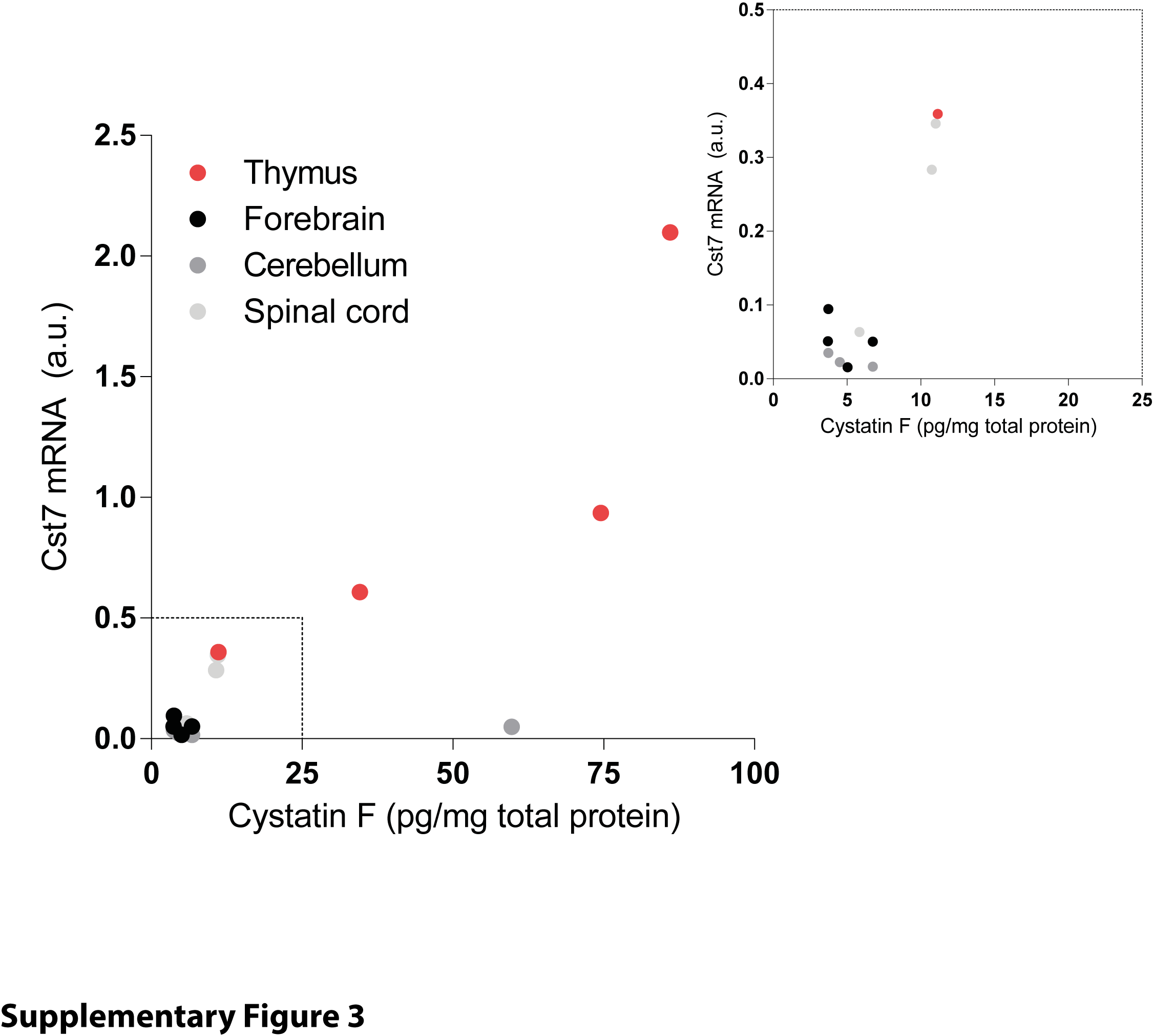

Supplement: S3 Fig — Correlation between cystatin F protein levels (x axis) and Cst7 mRNA levels (y axis) in thymus and different regions of the central nervous system (CNS) in adult C57BL/6J mice (n = 4 for all organs except n = 3 for spinal cord). Each dot denotes an individual organ or CNS area. Inset: magnification of the area delimited between the axis and the dashed lines. (TIF) [file pone.0171923.s003.tif]

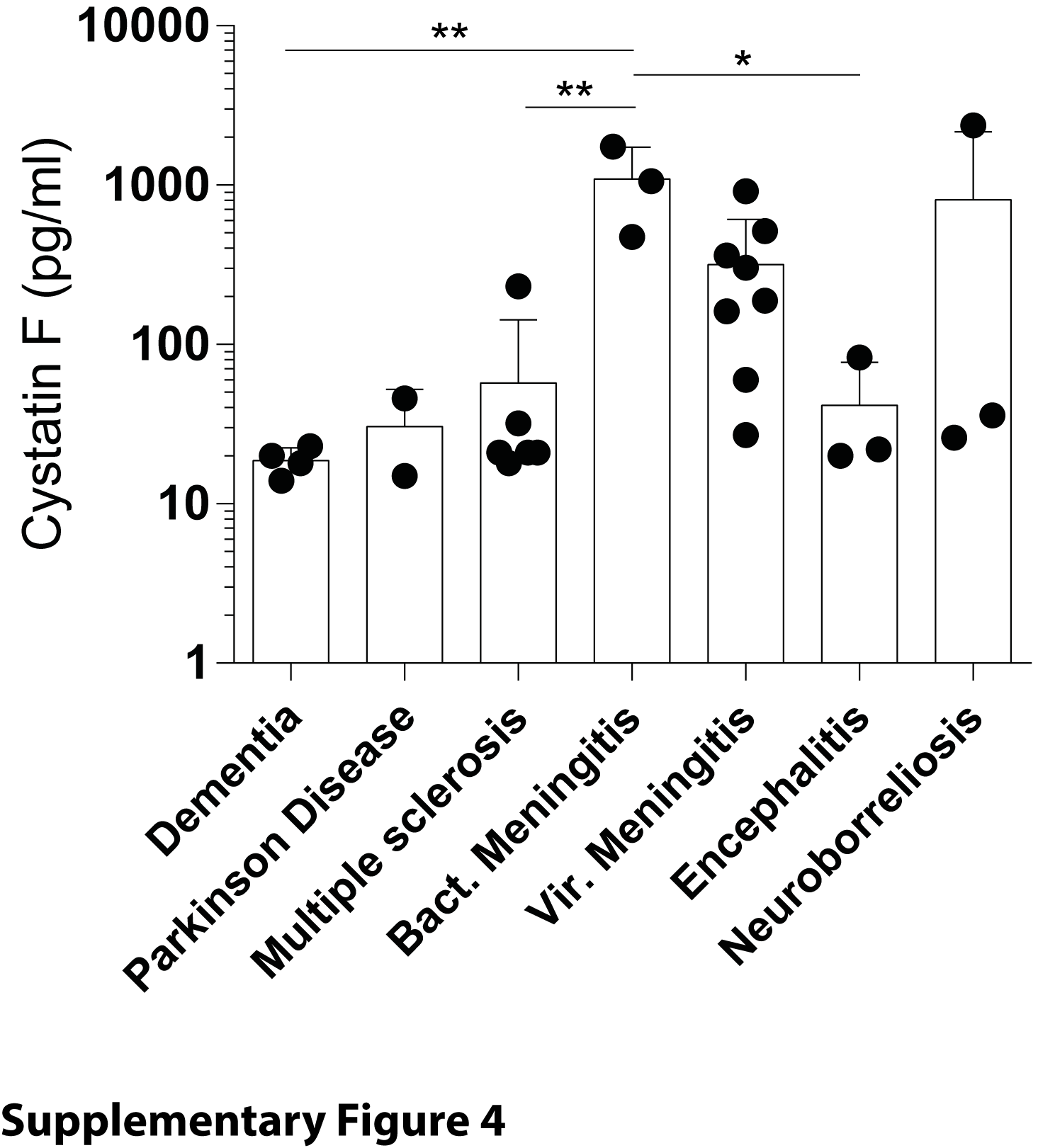

Supplement: S4 Fig — Cystatin F levels were measured in patients with different neurological conditions (Bact. Meningitis, bacterial meningitis; Vir. meningitis, viral meningitis). Dots: denote individual subjects; bars: mean; error bars: standard deviation (*, p<0.05; **, p<0.01, one-way ANOVA followed by Bonferroni’s multiple comparison test). (TIF) [file pone.0171923.s004.tif]

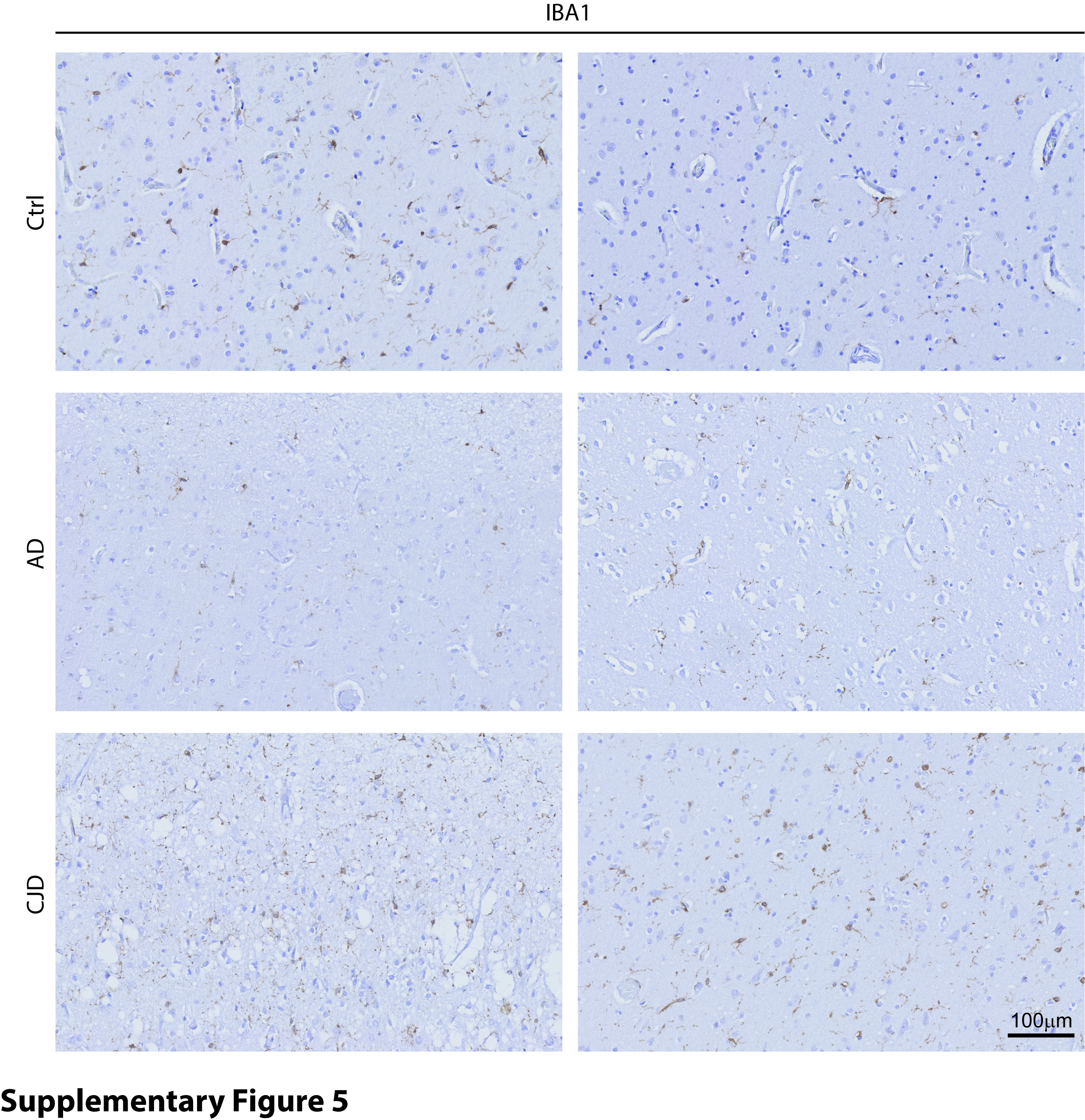

Supplement: S5 Fig — Histologic analysis of microglia (IBA1) in frontal cortex of the same cohort of patients with Alzheimer’s disease (AD) and Creutzfeldt-Jakob disease (CJD) reported in Fig 5. Non-demented subjects were included as control (Ctrl). Images from two representative cases per group are depicted. Scale bar: 100 μm. (TIF) [file pone.0171923.s005.tif]
