# Supplementary material for: Cystatin F is a biomarker of prion pathogenesis in mice
Source: PLoS One. 2017 Feb 8;12(2):e0171923. doi: 10.1371/journal.pone.0171923 (PMC5298286; doi:10.1371/journal.pone.0171923)
Supplement: S1 File — (PDF) [file pone.0171923.s006.pdf]

Cst7

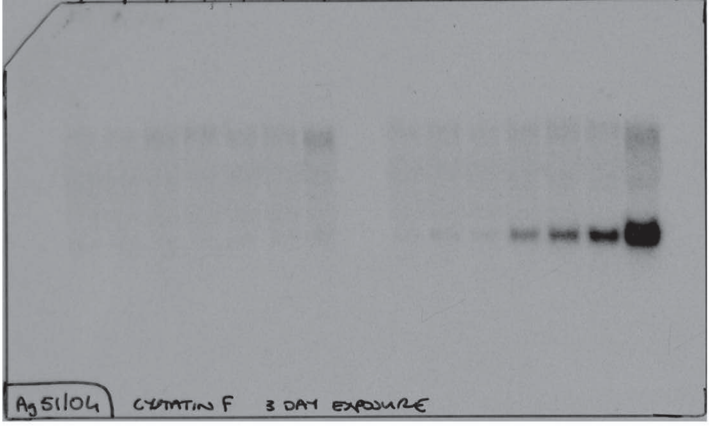

Serpina3n

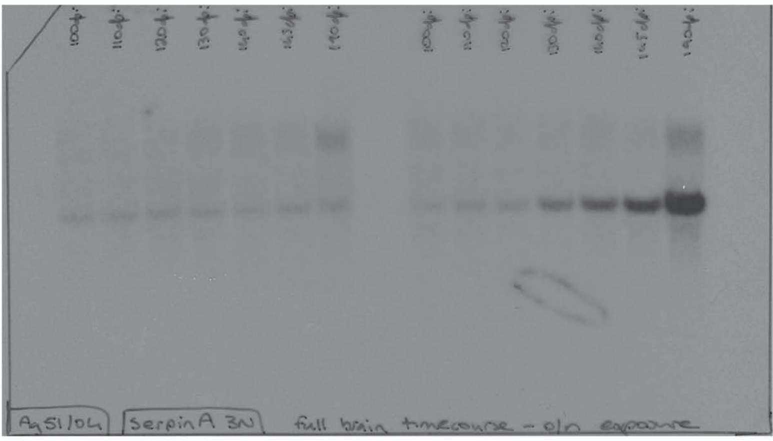

Gfap

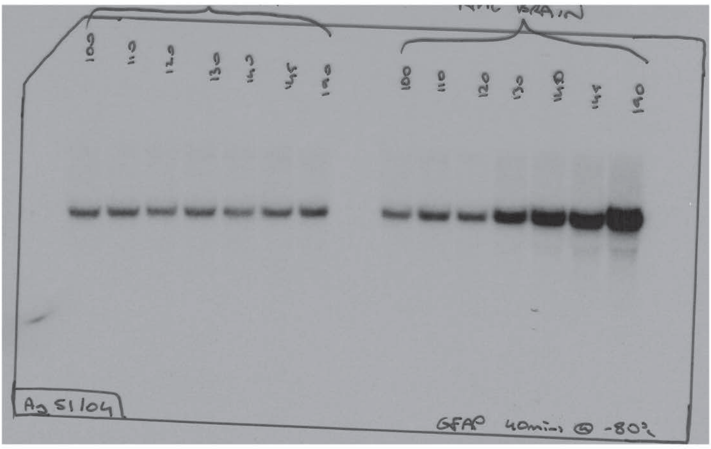

Rn18s

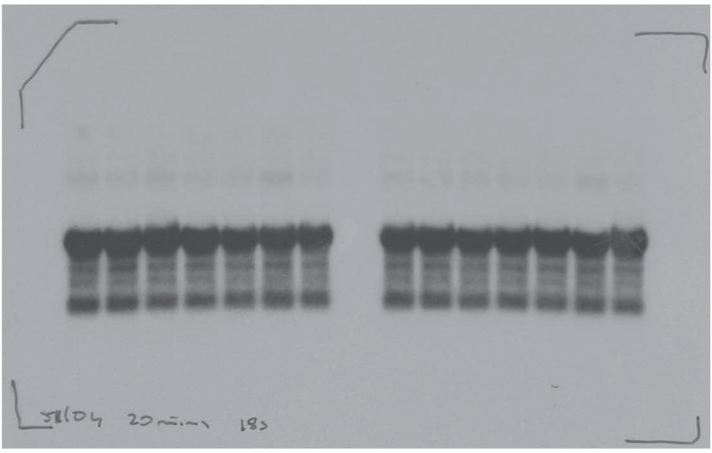

Uncropped images Fig 2A

Cst7

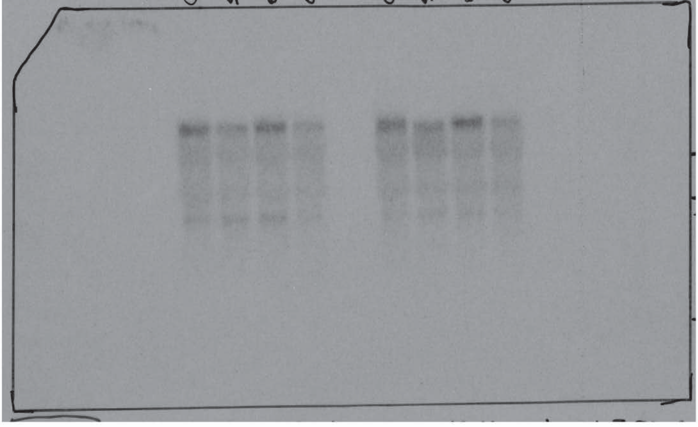

Serpina3n

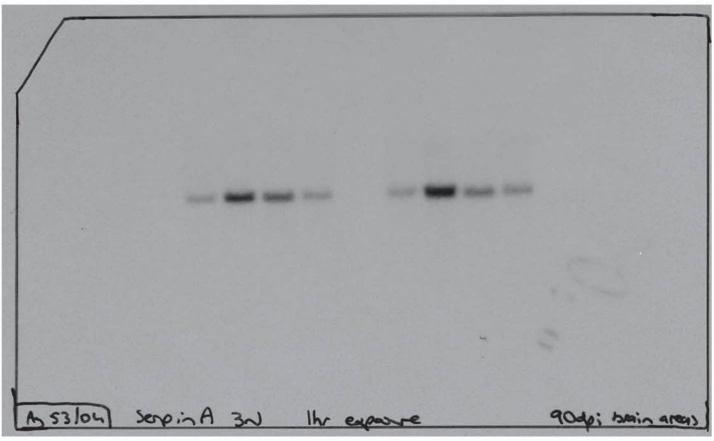

Gfap

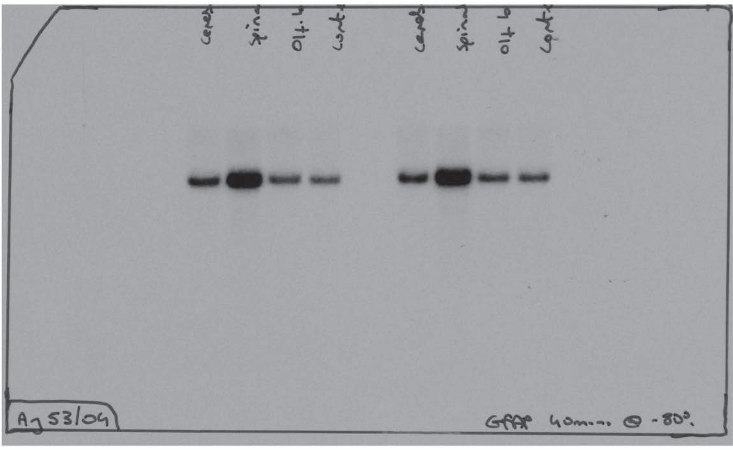

Rn18s

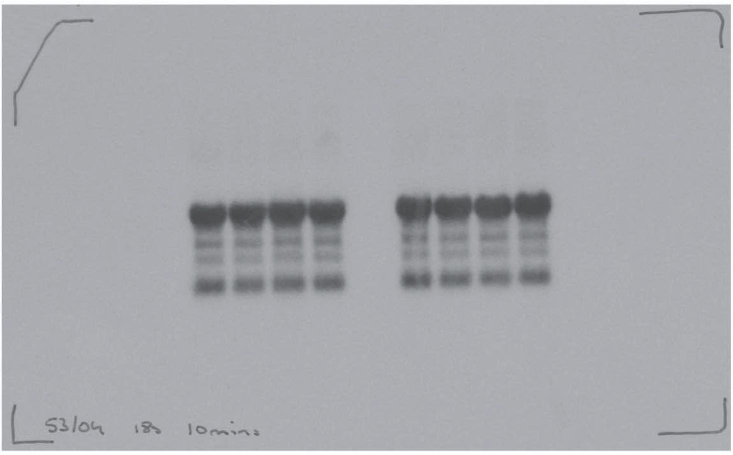

Uncropped images Fig 2B - 90 dpi panel

Cst7

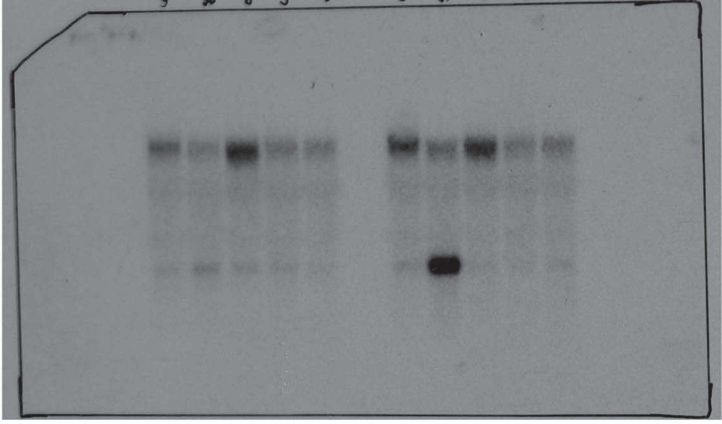

Serpina3n

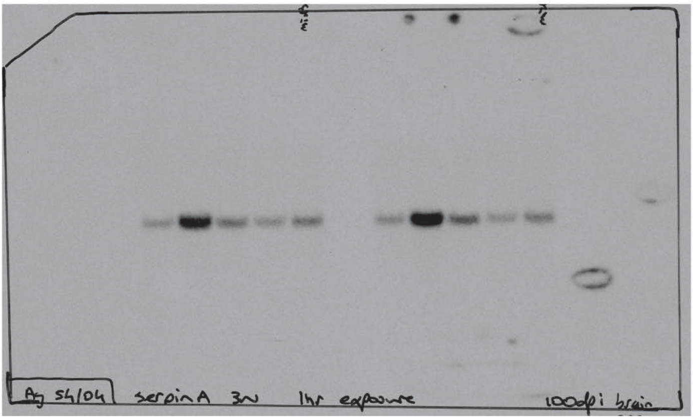

Gfap

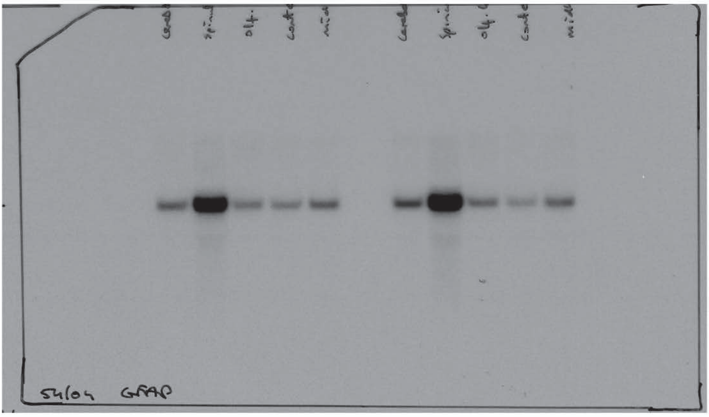

Rn18s

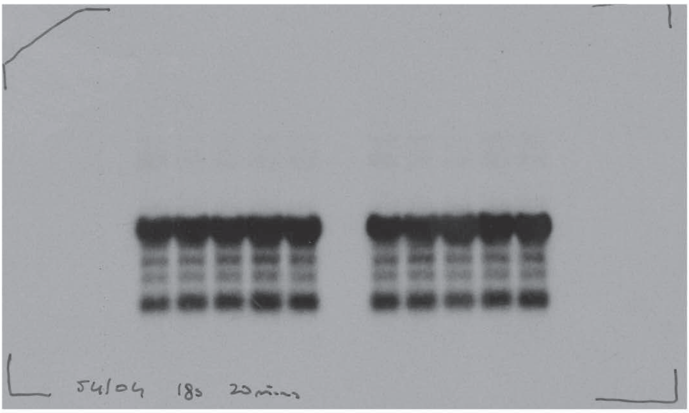

Uncropped images Fig 2B - 100 dpi panel

Cst7

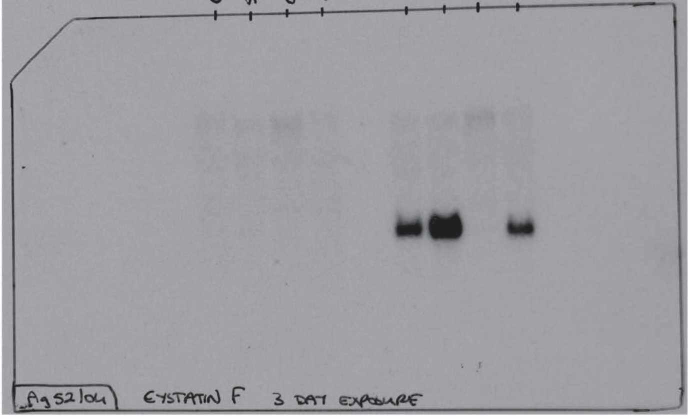

Serpina3n

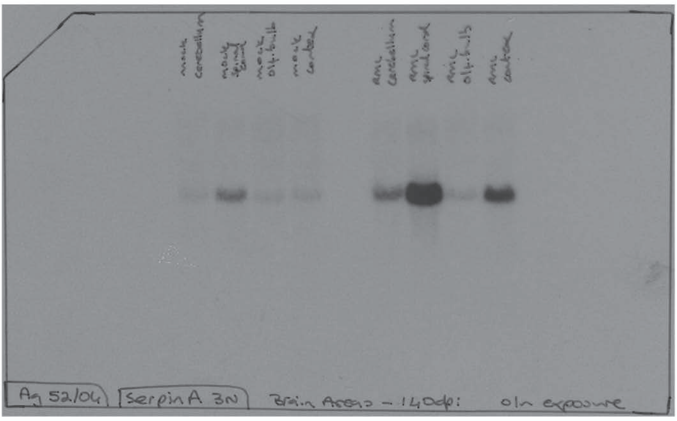

Gfap

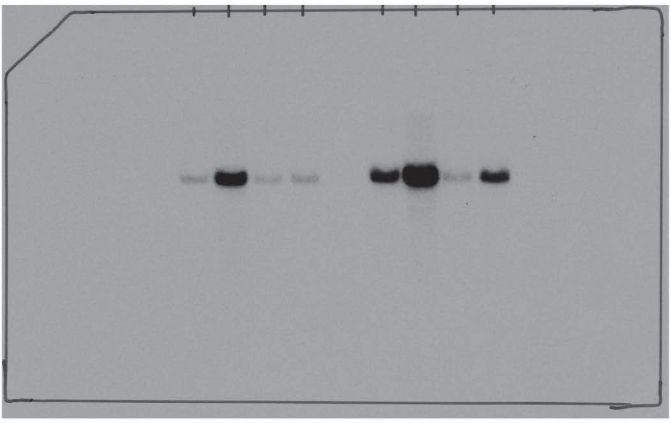

Rn18s

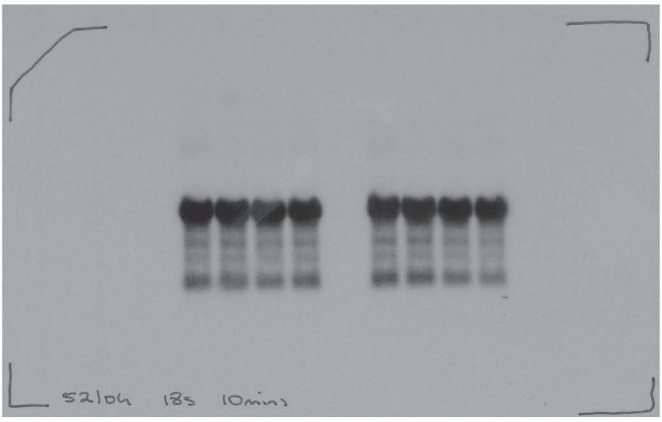

Uncropped images Fig 2B - 140 dpi panel

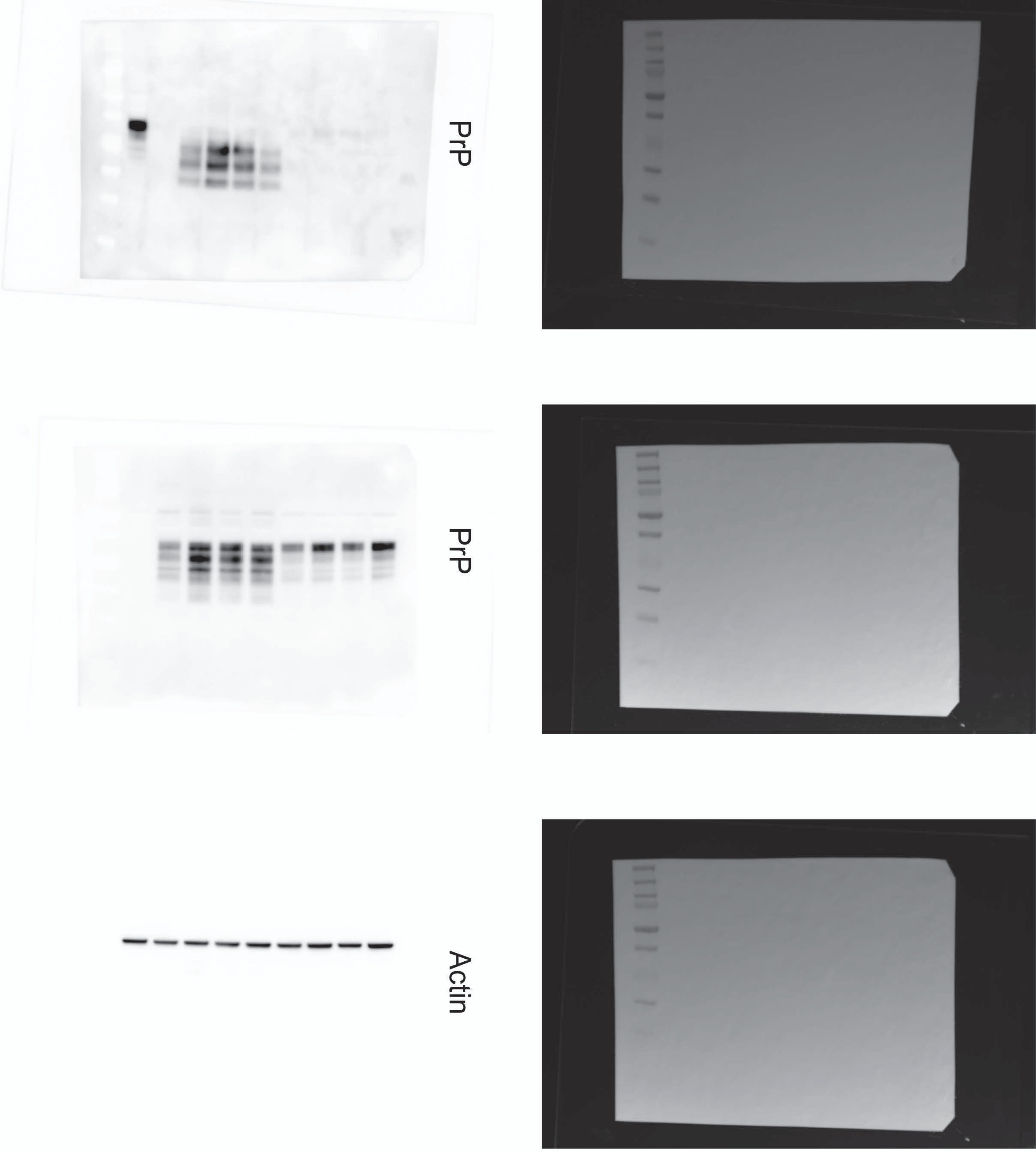

Uncropped images Fig 3

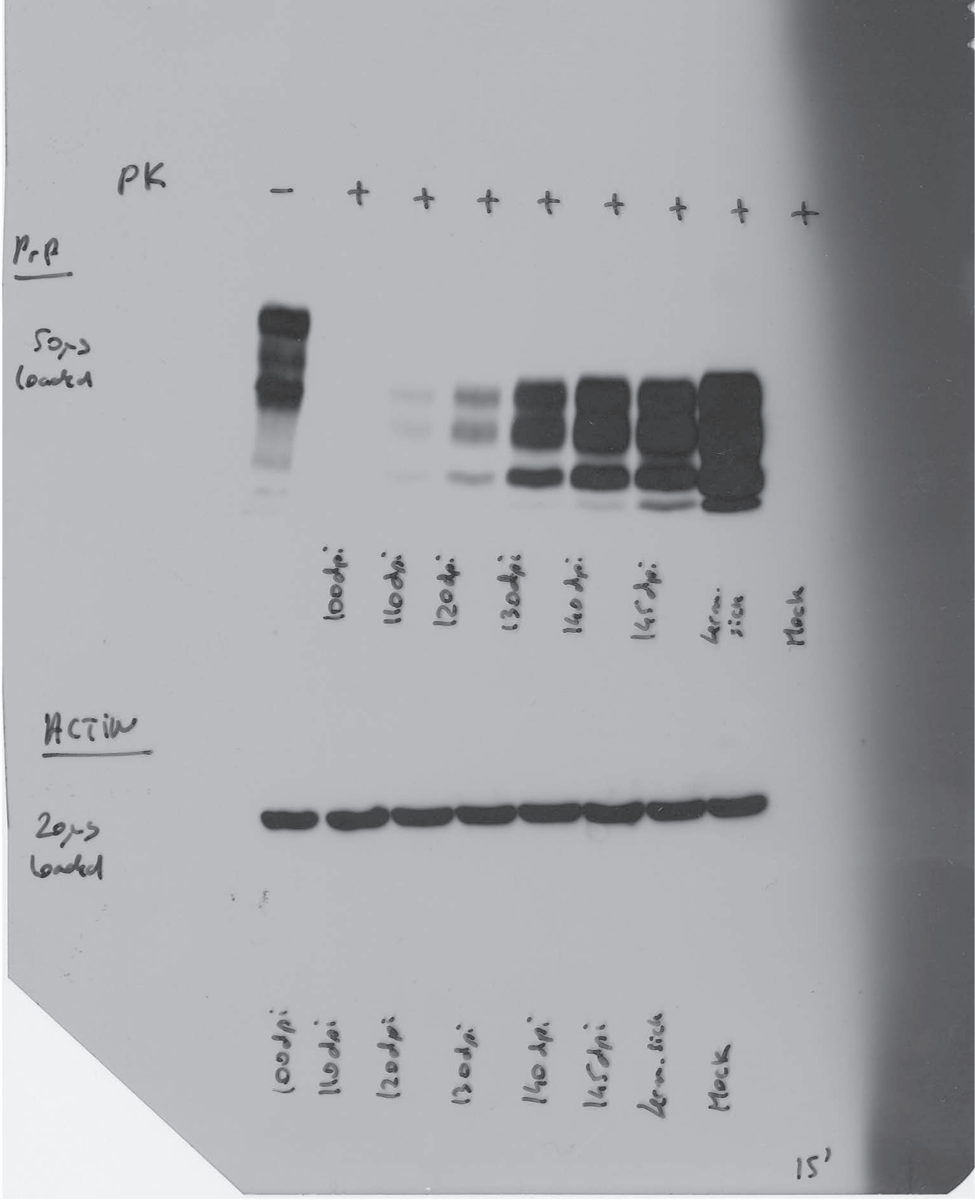

Uncropped images S1 Fig

Cst7

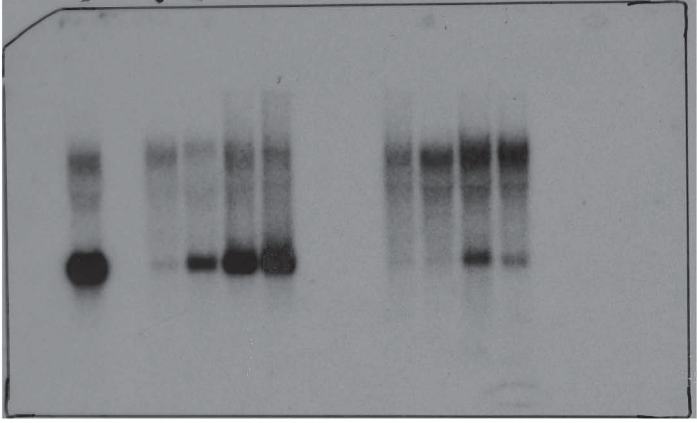

Serpina3n

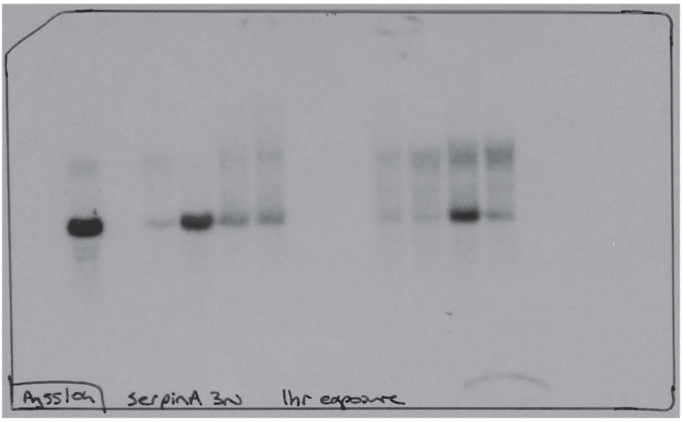

Gfap

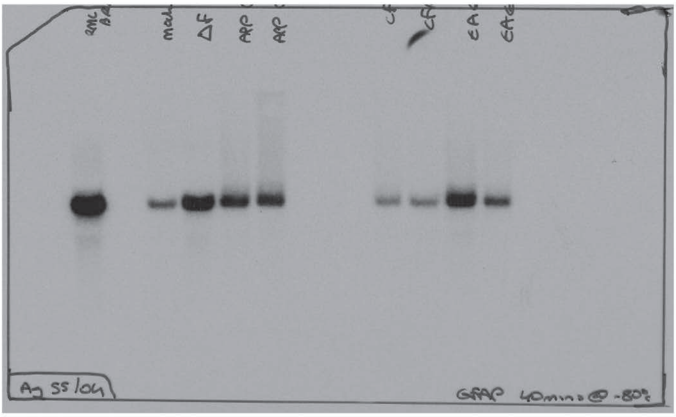

Rn18s

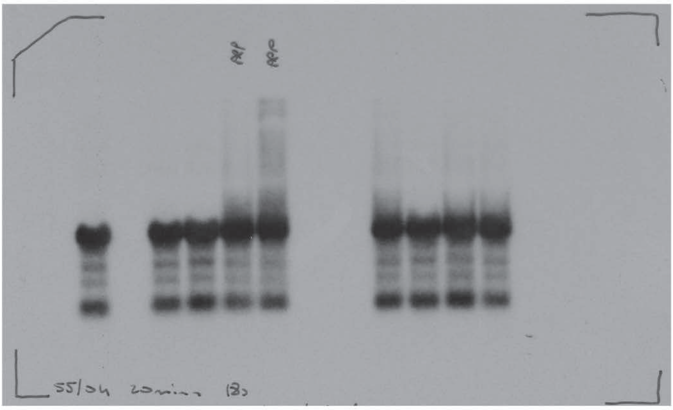

Uncropped images S2C Fig
