# Supplementary material for: Cystatin F is a biomarker of prion pathogenesis in mice
Source: PLoS One. 2017 Feb 8;12(2):e0171923. doi: 10.1371/journal.pone.0171923 (PMC5298286; doi:10.1371/journal.pone.0171923)
Supplement: S1 Table — (DOCX) [file pone.0171923.s007.docx]

**S1 Table. Clinical assessment and scoring of wild-type mice inoculated with RML prions**

After inoculation, mice were observed three times per week for clinical signs including gait, grooming, activity, rough hair coat, limb paresis and ataxia. After the appearance of the first sign of scrapie (grade 1), mice were monitored every day and wet food was supplied in the cage. When mice reached score grade 2 that hamper the mice reaching water bottle, they were euthanized.

| **Score** | **Clinical signs** | **Assessment** | **Action** |
| --- | --- | --- | --- |
| 0 | No detectable signs of abnormal movement |  |  |
| 1 | Waddling gait, mild signs of reduced grooming, rough hair coat, limb weakness, front leg paresis* | Slight rolling while shaking the cage | Provide wet food in the cage;  Observe every day |
| 2 | Ataxia, reduced grooming and activity, paralysis, rolling* | Rolling while shaking the cage | Euthanize immediately if the clinical signs hamper the mice reaching the water bottle |
| 3 | Dead |  |  |

Modified from Zhu C et al. Unaltered Prion Pathogenesis in a Mouse Model of High-Fat Diet-Induced Insulin Resistance. PLoS One 2015.
