# Supplementary material for: Cystatin F is a biomarker of prion pathogenesis in mice
Source: PLoS One. 2017 Feb 8;12(2):e0171923. doi: 10.1371/journal.pone.0171923 (PMC5298286; doi:10.1371/journal.pone.0171923)
Supplement: S2 Table — (DOCX) [file pone.0171923.s008.docx]

**S2 Table. Clinical assessment and scoring of wild-type mice after induction of experimental autoimmune encephalitis**

Mice are initially observed four times per week and then daily from the onset of clinical signs. After onset of EAE symptoms mice are scored at least every second day (daily if clinical score of ≥3 the mice and until remission below score 3).

| **Score** | **Clinics** | **Assessment** | **Action** |
| --- | --- | --- | --- |
| 0 | No detectable signs of EAE |  |  |
| 0.5 | Distal limp tail |  |  |
| 1.0 | Complete limp tail |  |  |
| 1.5 | Limp tail and hind limb weakness | Occasional grid test positive | From now on provide feed and water in the cage (separate) |
| 2.0 | Unilateral partial hind limb paralysis | One leg consistently falls through the grid (cage test) |  |
| 2.5 | bilateral partial hind limb paralysis | Cage grid test bilaterally positive |  |
| 3.0 | Complete bilateral hind limb paralysis |  | Sacrifice if persistent  >7 days |
| 3.5 | Complete bilateral hind limb paralysis and unilateral forelimb paralysis |  | Sacrifice if persistent  >3 days |
| 4.0 | total paralysis of fore and hindlimbs |  | Sacrifice immediately |
| 5.0 | dead |  |  |

Modified from Becher B et al. The clinical course of experimental autoimmune encephalomyelitis and inflammation is controlled by the expression of CD40 within the central nervous system. J Exp Med 2001.
