# Supplementary material for: Cystatin F is a biomarker of prion pathogenesis in mice
Source: PLoS One. 2017 Feb 8;12(2):e0171923. doi: 10.1371/journal.pone.0171923 (PMC5298286; doi:10.1371/journal.pone.0171923)
Supplement: S3 Table — (DOCX) [file pone.0171923.s009.docx]

| **Gene** | **Accession** | **Forward primer (5'-3')** | **Reverse primer (5'-3')** |
| --- | --- | --- | --- |
| *Actb* | NM007393 | ATGGATGACGATATCGCTG | ATGAGGTAGTCTGTCAGGT |
| *Aqp4* | U48399 | AGCAATTGGATTTTCCGTTG | TGAGCTCCACATCAGGACAG |
| *B2m* | NM009735 | ACACTGAATTCACCCCCACT | AAAGAAGGTGATGTGTACATTGCT |
| *C1qa* | NM007572 | CACCAACCAGGAGAGTCCAT | AAGATGCTGTCGGCTTCAGT |
| *C1qc* | AW227993 | GTTCAACAGCAAGCAGGTCA | AGGAACCAGGGTGGACTTCT |
| *C4b* | NM009780 | TGCCTTCCGTCTCTTTGAGT | TGAAGGCATCTCCTCAATCC |
| *Cd22* | NM009845 | TGGATGGAGCCCATTCACCT | TTCCGTGGTCCGTCCACTTT |
| *Cebpd* | BB831146 | CTTCACCAGGGAAAAGCAAG | CTTCGGCAACCACCTAAAAG |
| *Chil1* | BC005611 | CGCAGCTTTGTCAGCAGGAA | GGTGGGGATGCCCATCAGTA |
| *Cst7* | NM009977 | CGTGTTTTCCCCACTCCTTA | CTCTGCACGTGCTCCAGTAA |
|  |  | CCTGCCTTGAAGCGGACTC | CAGGCACCTCAAAACTGTGG |
| *Ctsh* | NM007801 | CAACTGATTGGCAGACCAAG | TCTAGGTCCACGTGGGTTTG |
| *Ctss* | NM021281 | TAAAGGGCCTGTCTCTGTGG | GCAATTCCGCAGTGATTTTT |
| *Eif2a* | NM001005509 | CAACGTGGCAGCCTTACA | TTTCATGTCATAAAGTTGTAGGTTAGG |
| *Fabp7* | NM021272 | GAAGGTGGCAAAGTGGTGAT | ACAGCAACGATATCCCCAAA |
| *Fcgr1* | AF143181 | CATATAGCAAGGGCGGAAAG | ACACGCCATCGCTTCTAACT |
| *Fcrls* | BC016551 | GCACAATCGTGAACATCACC | ATGCCTTTCCTCCAAAAGGT |
| *Gapdh* | NM008084 | TCCATGACAACTTTGGCATTG | CAGTCTTCTGGGTGGCAGTGA |
| *Gfap* | BB183081 | AAGGGACCATTCCCTGTCTT | AGGTTAGCGGAGGTGGAAAG |
| *Grn* | AV166504 | AAGGTTGGGAATGTGGAGTG | TTCCCAGGACTGTGGAGTTC |
| *H2-D1* | L36068 | GGAAAAGGAGGGGACTATGC | TTGGCTATGGAAGGGAACAC |
| *H2-K1* | BC011306 | TTCCTCCATCCACTGTCTCC | CTGTCACCAAGTCCACTCCA |
| *H2-L* | M34962 | ACATGGAGCTTGTGGAGACC | CTGGAGCCAGAGCATAGTCC |
| *Hprt1* | NM013556 | TCCTCCTCAGACCGCTTTT | CCTGGTTCATCATCGCTAATC |
| *Ly86* | NM010745 | CTGATGGCAAAAGGCTCTTC | GAGGAGGTGACAGTGGCATT |
| *Mpeg1* | L20315 | AACCTGCCTCTTGAGCGTTA | TTCTGATGTGCCTTGCTTTG |
| *Nupr1* | NM019738 | CACCAACAGCCAACCCTTCC | CCATTGCTGGGTGTGGTGTC |
| *Plek* | AF181829 | ACTTTGGCAAACGGATGTTC | GATACAAAGCCCCCAAGTCA |
| *Ptprc* | NM011210 | CCACCAGGGACTGACAAGTT | TGTAATTTGTTTGGGCACGA |
| *Serpina3n* | NM009252 | TATCTGCCTCCACCCAAAAG | GCCAGATGTGGACAAAGTGA |
| *Sgk1* | NM011361 | AGAGCACACCCTCCTCTCAA | TCAGAAAAGGCACATTGCAG |
| *Slc25a18* | AK005250 | GGACATTCCCTTCTCCATCA | CTCCTGTGTCCAGAGCTTCC |
| *Smc4* | BI665051 | ACACAGGGGCTTCAGAAAGA | CCTTGATTGCAGCTTTCCTC |
| *Tgfbr1* | BM248342 | TCCACCAGGTTTGCAATTTT | AAACTGATGGGGAAATGCAG |
| *Utp6c* | NM144826 | TTTCGGTTGAGTTTTTCAGGA | CCCTCAGGTTTACCATCTTGC |

**S3 Table. List of primers used in this study**

*Eif2a*, *Hprt1* and *Utp6c* primer sequences from Kosir et al., BMC Mol Biol 2010; *Gapdh* primer sequences from Genomics Platform, University of Geneva.
